# Plant influence shapes *Andropogon gerardii* rhizobiome assembly strategies in response to decreasing precipitation

**DOI:** 10.1128/spectrum.02832-25

**Published:** 2026-07-15

**Authors:** Brooke Vogt, Anna Kazarina, Jack Sytsma, Bryttan Adams, Leslie Rodela, Reece Keller, Darcy Thompson, Helen Winters, Ari Jumpponen, Loretta Johnson, Sonny T. M. Lee

**Affiliations:** 1 Division of Biology, Kansas State University 167079, Manhattan, Kansas, USA; Instituto de Ecología, A.C. (INECOL), Pátzcuaro, Michoacán, Mexico

**Keywords:** microbiome, microbial community assembly, plant-microbe interactions, climate change, 100^th ^meridian

## Abstract

Predicted changes in precipitation threaten tallgrass prairies by altering microbial communities essential for plant resilience. *Andropogon gerardii*, a dominant tallgrass, spans the North American precipitation gradient. However, it remains unclear to what extent the rhizosphere microbiomes (rhizobiomes) are influenced by the plant-host-environmental interaction. To assess how environmental and host influences shape the rhizobiome, we surveyed *A. gerardii* populations across 25 remnant prairie sites within its native range in the United States, characterizing the microbiomes in the rhizosphere and soils using 16S amplicon sequencing. We demonstrated that while geographic location was the primary driver of community structure of both rhizosphere and soil communities, regional precipitation emerged as an influential determinant of the microbial community assembly. We observed distinct microbial divides across the dry and wet regions of the North American “arid-humid divide.” Importantly, we found compelling large-scale evidence that regional precipitation has a profound influence on rhizobiome assembly. In the most arid regions, rhizosphere microbial communities exhibited significantly greater compositional convergence than local soil communities and harbored distinct, enriched sub-populations of taxa previously associated with host-benefiting functions. Our study aims to lay the groundwork for future investigations into the functional pathways through which limited precipitation shapes rhizobiome assembly, and how these effects are further impacted by host influence and local adaptation.

**IMPORTANCE:**

In this study, we conducted a biogeographical survey of the native tallgrass species *Andropogon gerardii* across 25 remnant prairie sites spanning the contiguous United States. Using 16S rRNA amplicon sequencing, we analyzed microbial communities from both local soils and plant-associated rhizobiomes. Our results demonstrate that regional precipitation is an influential driver of microbial community assembly, with clear compositional shifts observed across the 100^th^ meridian, the North American “arid-humid divide.” Critically, in the most arid locations, rhizobiomes converged toward a more homogeneous community structure, with both βNTI and RC_Bray_ indicating more deterministic assembly, consistent with intensified selective filtering under limited precipitation. These patterns establish a framework for future work to dissect the specific host and microbial mechanisms that enforce deterministic community assembly under precipitation stress.

## INTRODUCTION

Changes in the assembly and function of plant host-associated microbiomes can be influenced by a mosaic of factors, including abiotic stress induced by climate change (1–4). Over the past 10,000 years, the dominant tallgrass prairie grass, *Andropogon gerardii*, has diverged into genetically distinct ecotypic varieties based on precipitation (dry, mesic, and wet ecotypic varieties) across North America (5–9). Microevolutionary processes have modified the morphological and physiological traits of these varieties, enabling them to survive and thrive in regions with varying precipitation levels, temperatures, and soil physicochemical properties (6, 10–12). These adaptations have resulted in distinct geographic ranges for these historically well-documented (6, 8, 10, 12, 13) host varieties, each aligning with their respective sub-regions along the 100^th^ meridian, the North American “arid-humid” divide (14). For example, in a decade-long reciprocal garden experiment across a precipitation gradient from western Kansas to southern Illinois, each host variety demonstrated the highest cover and biomass coupled with specific transcriptomic profiles at its respective home location, providing clear evidence of local adaptation (15). Additionally, when rainfall is reduced via rainout shelters, the dry variety proves better adapted to water limitation, in some cases even improving performance under enhanced drought (15). While local adaptation has been well studied, the extent to which *A. gerardii* structures its microbiomes remains largely unresolved. If such an influence exists, we predict that the rhizobiome composition would likely mirror the geographic boundaries and ecological differentiation of its host variety.

Previous studies have reported conflicting evidence regarding the relative roles of host identity and local environment in shaping rhizobiome communities. Some research suggests that rhizobiome composition is primarily determined by local abiotic and edaphic conditions, resulting in distinct microbial communities even among plants of the same variety at different locations (16, 17). This perspective emphasizes strong environmental filtering and suggests minimal host control. Conversely, other studies propose that rhizobiomes of the same host variety are similar across locations and differ markedly from those of other varieties (18, 19). This view implies that host-mediated selection under precipitation-driven stress recruits similar microbial communities within the same host variety, overriding local environmental variation (20, 21).

Prior reciprocal garden experiments (22, 23), examining the rhizobiomes of the dry, mesic, and wet varieties of *A. gerardii,* have supported the idea that host-associated rhizobiomes are primarily influenced by regional precipitation. In these studies, *A. gerardii* recruited rhizobiomes similar to those at its native sites, including “specialist” microbial populations, supporting the “home-field advantage” hypothesis (22, 23). While our previous experiments provided insights into the *A. gerardii* “home-field advantage” hypothesis, how long-term variation in precipitation across the species’ geographic range shapes plant-rhizobiome relationships remains poorly understood.

While the differences among dry, mesic, and wet varieties of *A. gerardii* have evolved over thousands of years due to natural selection, it remains to be seen how quickly their rhizobiome can adapt to long-term changes in precipitation. The consequences of a rhizobiome’s ability to adjust to different forms of precipitation-based abiotic stress are crucial. In this century, the 100^th^ meridian has shifted eastward, a trend predicted to accelerate due to the compounding effects of climate change (5, 24–27). Understanding the host rhizobiome assembly and its vulnerabilities is crucial, mainly because *A. gerardii* constitutes up to 80% of the biomass in tallgrass prairies (28). In addition, central tallgrass prairies are critically endangered, with only 4.4% remaining in North America (6, 8, 28–31). Rapid shifts in precipitation patterns may lead to a decline in *A. gerardii* if their microbiomes are closely linked to local ecological and soil microbial communities, and cannot adapt to long-term precipitation shifts. If *A. gerardii* microbiomes are solely influenced by local conditions, then long-term variations in rainfall could pose a significant threat to the host’s health, longevity, and survival. However, it is also possible that the host exerts a considerable influence on its rhizobiome, buffering the effects of environmental change. We hypothesized that the influence of plant host identity and environmental filtering on rhizobiome assembly varies along the precipitation gradient (32, 33). Specifically, we predicted that in arid, high-stress environments, host identity played a stronger role, promoting more uniform, host-specific rhizobiomes enriched in specialist taxa and more distinct from those found in local soils. In contrast, we predicted that under low-stress precipitation conditions, environmental filtering dominated, leading to higher stochasticity, greater similarity between soil and rhizobiome communities, and a prevalence of generalist taxa.

To address our hypotheses and understand the composition of microbial communities associated with *A. gerardii*, we conducted a biogeographical survey at 25 locations across the contiguous United States. We analyzed 16S rRNA amplicon data from local soil (LS) and plant host-associated rhizobiome (RB) samples. Our study addressed three key questions: (i) How do local and regional characteristics influence microbial community composition? (ii) To what extent does plant host selection appear to shape rhizobiome composition and diversity relative to local soil, and is there evidence that biotic filtering varies across different abiotic stress conditions? (iii) How do local soil and rhizobiome communities compare in similarity across different precipitation-related host variety ecotypes? Our study aims to lay the groundwork for future research by clarifying how host genetic diversity and environmental pressures together shape rhizobiome assembly and function.

## MATERIALS AND METHODS

### Sample collection and preparation

Sample collection took place between June and August of 2023. We selected 25 sample locations based on the historical distribution of *A. gerardii*, aiming to represent variations in precipitation (34), soil physicochemical properties, climate, and host variety across the contiguous United States (Table S1). We collected local soil and rhizosphere samples from six haphazardly chosen *A. gerardii* plants at each location. From each plant, we sampled one soil core (10 cm deep and 1.25 cm in diameter) near the base of the plant tillers. This core sample was manually homogenized in a sterile plastic bag. From the homogenized sample, three aliquots of roots (approximately 20 cm in length each) were manually collected, gently shaken to remove any loose soil, and placed into three microcentrifuge tubes. From the remaining soil, we collected three soil aliquots (~1 g each) into three microcentrifuge tubes. In summary, at each of the 25 locations, we collected both local soil and rhizosphere samples, with six biological replicates taken from six different *A. gerardii* individuals. Each biological replicate was analyzed in triplicate, resulting in a total of 900 samples overall (36 per site; 450 per sample type). Any remaining soil (without roots) from the six plant replicates that were not used for the local soil or rhizobiome samples was stored in sterile bags on ice for the soil physicochemical property analyses (*n* = 25 samples). All samples were stored on ice in a cooler until they were frozen at −80°C within 96 h after sampling for further processing.

### Soil physicochemical properties

The soil physicochemical property analyses were conducted at Kansas State University’s Soil Testing Laboratory (https://www.agronomy.k-state.edu/%C2%A0outreach-and-services/soil-testing-lab/soil-analysis/researchers.html). The analyses were performed on the local soil samples (*n* = 25). Detailed procedures are in the revised version of the *Recommended Chemical Soil Test Procedures for the North Central Region* manual (35, 36). However, a summary of the methods for each test is provided below.

Samples were analyzed for various physicochemical properties, including soil pH, total nitrogen, carbon content (both organic and inorganic), phosphorus content, and texture (percentages of sand, silt, and clay). Before analysis, the samples were dried in a drying oven at 60°C overnight. The dry soils were passed through a 2 mm sieve to break up aggregates and ensure homogeneity.

For pH analysis, soil was mixed with an equal amount of deionized water in a 1:1 solution (35, 37–39). The mixture was stirred for 5 s or until a uniform soil-water slurry was achieved. After allowing it to sit for 10 min, pH measurements were taken using a Skalar SP50 Robotic Analyzer (Skalar Inc.; Georgia, USA).

Phosphorus concentrations were measured in parts per million (ppm P) using the Mehlich III method on 2 g of soil (35, 40, 41). The soil was suspended in a 1:10 solution with the standard Mehlich III solution, which consists of 0.2 N acetic acid (CH₃COOH), 0.25 N ammonium nitrate (NH₄NO₃), 0.015 N ammonium fluoride (NH₄F), 0.013 N nitric acid (HNO₃), and 0.001 N EDTA (ethylenediaminetetraacetic acid) (HOOCCH_2_)_2_NCH_2_CH_2_N(CH_2_COOH)_2_). The mixture was shaken at 200 excursions per minute for 5 min. After shaking, the slurry was filtered through a Whatman No. 42 filter. From the clear filtered slurry, 2 mL was transferred to a test tube, and 8 mL of the working solution was added. The working solution was prepared by combining 25 mL of Murphy-Riley Solution A (which contains 0.051 N ammonium molybdate [(NH₄)₆Mo₇O₂₄·4H₂O] and 0.004 N antimony potassium tartrate [K₂Sb₂(C₄H₂O₆)₂·3H₂O]) with 10 mL of Solution B (0.749 N ascorbic acid [C₆H₈O₆]) in a total volume of 1 L.

After letting the mixture sit for 10 min, the samples were loaded in triplicate onto a 96-well plate and analyzed using a Lachat QuikChem 8500 Series 2 photometric colorimeter (Hach Inc.; Colorado, USA) at a wavelength of 882 nm, following the orthophosphate method (12-115-01-1-A). Phosphorus measurements were determined by comparing the samples against a 500 ppm potassium dihydrogen phosphate (KH₂PO₄) stock solution, diluted to a 50 ppm standard solution before loading into the 96-well plate. Exchangeable potassium (K^+^) was determined using a neutral ammonium acetate extraction (35). Briefly, 2 g of air-dried, sieved soil were extracted with 20 mL of 1 M ammonium acetate (NH_4_OAc, [pH = 7.0]) at a 1:10 soil to solution ratio (35). The soil samples were then shaken for 5 min at 200 excursions per minute and subsequently filtered through a Whatman No. 2 filter paper (35). Potassium concentrations in ammonium acetate soil extracts were quantified by inductively coupled plasma–optical emission spectroscopy (ICP-OES) at 766 nm using an Agilent 5800 ICP-OES (Agilent Technologies, Santa Clara, CA, USA). Concentrations were determined using external potassium calibration standards prepared in 1 M ammonium acetate and were reported as ppm K^+^.

Total nitrogen and total carbon (including organic and inorganic components) analyses were conducted on 0.35 g of soil using a LECO TruSpec CN analyzer (LECO; Michigan, USA), following the procedures outlined in the operation manual (42). The samples were encapsulated and securely sealed in the loading head. They were then purged to eliminate any atmospheric gases that could interfere with the measurements. Once all atmospheric gases were removed, the samples were combusted at 950°C to ensure rapid combustion while flushed with pure oxygen. A consistent aliquot of gases was then homogenized and separated. The total percentage of carbon and nitrogen was calculated based on weight percentages using the formula: Total % of Carbon or Nitrogen = (Mass of C or N / Total Sample Mass) × 100. The analysis showed a standard deviation of 2.18% for soil carbon and 3.35% for soil nitrogen.

Soil texture was determined using a modified hydrometer method (43, 44) on a 50 g soil sample. First, the soil was placed in a dispersion cup, and then 100 mL of a 5% sodium hexametaphosphate solution (Na_6_(PO_3_)_6_) was added. The mixture was stirred for 30 to 60 s until uniform. This slurry was then transferred to a 1,000 mL cylinder filled to the 1,000 mL mark with deionized water. The mixture was allowed to settle overnight. To begin the analysis, a plunger was inserted, and the sample was mixed for an additional 30 s to ensure homogeneity. A Bouyoucos hydrometer (Fisherbrand; Soil Analysis ASTM Hydrometer) was then placed in the slurry. After 40 s, the initial hydrometer reading was recorded. A second reading was taken after 2 h.

Percentages of sand, silt, and clay were calculated as follows: clay (%) was calculated as (final hydrometer reading × 100) divided by the oven-dry mass of the soil sample; silt (%) was calculated as (initial hydrometer reading × 100) divided by the oven-dry mass of the soil sample; and sand (%) was calculated by difference as 100 − silt (%) − clay (%). All readings were adjusted using a blank sample (100 mL of the 5% dispersion solution plus 880 mL of deionized water) and applying temperature and density corrections. For every one °F above 68°F, 0.2 was added to the reading, while for every one °F below 68°F, 0.2 was subtracted from the reading.

### DNA extraction, 16S rRNA amplicon sequencing, and analyses

We extracted eDNA from the samples using the E.Z.N.A. Soil DNA Kit (Omega BIO-TEK; Georgia, USA), following the manufacturer’s protocol with minimal modifications (45). The only adjustment for the local soil samples was the use of glass beads for cell lysis, performed with a Qiagen TissueLyser II (Qiagen; Hilden, Germany) at 30 revolutions per second for 10 min. For each of the 25 locations, we extracted three aliquots separately and selected one from each of the six plants based on DNA concentration and quality. The final elution volume for each local soil sample (*n* = 150) was 100 μL.

Similar to the soil samples, we extracted eDNA from the rhizosphere samples using the E.Z.N.A. Soil DNA Kit (Omega BIO-TEK; Georgia, USA) with significant modifications to the manufacturer’s protocol to ensure optimal DNA quality and yield. In summary, for these samples, we combined three aliquots into one during the binding phase of the protocol. Additionally, we used liquid nitrogen to physically macerate the roots into a fine powder and then lysed the tissues using the TissueLyser II for 16 min at 30 revolutions per second. Depending on the sample location, we adjusted the final elution volumes for the rhizobiome samples, ranging from 30 μL to 150 μL.

We used the Qubit dsDNA Broad-Range (BR) DNA quantification kit on a Qubit 4 Fluorometer (Thermo Fisher Scientific, Invitrogen; Massachusetts, USA) to determine the sample eDNA concentration. The quality of the eDNA was assessed by measuring the A260/A280 and A260/A230 ratios on a Nanodrop One Spectrophotometer (Thermo Fisher Scientific, Invitrogen; Massachusetts, USA). After quantifying the eDNA, we normalized each sample to a concentration of 5 ng/μL using molecular-grade DNA/RNA-free water.

The normalized eDNA was sequenced at the Kansas State University Integrative Genomics Facility (https://www.k-state.edu/igenomics/). The eDNA amplification was performed using the 515F and 806R primers (46) (16S rRNA v4 amplicon region) and sequenced with 2 × 300 cycles on the Illumina MiSeq (Illumina; California, USA). We utilized QIIME 2 v. 2024.5 (47) to process the raw reads. We applied the QIIME 2 cutadapt (48) plugin to remove the primer sequences.

Subsequently, we eliminated sequences with an error rate greater than 0.1 and those with undetermined bases or without a primer. For further quality control, we used DADA2 (49) with consistent parameters across various runs to trim the reads, ensuring that the 25^th^ percentile of reads had a quality score above 15 (Forward = 270 and Reverse = 250). To mitigate biases caused by differences in sequence depth across samples in subsequent diversity analyses, we rarefied the data set to 15,000 reads per sample. We used the QIIME 2 pipeline to compute alpha (Shannon’s entropy [H'], Faith’s phylogenetic diversity [Faith’s PD], and number of observed features [*S*_obs_]) and beta diversity metrics. Finally, we employed the pre-trained classifier in QIIME 2 for bacterial taxonomic classification, referencing the SILVA v. 138 and 138.1 (βNTI and oligotyping analyses only) databases (50). After generating the ASV table, we removed all ASVs assigned to Eukarya, chloroplasts, or mitochondria, as well as those that remained unresolved or unclassified at the domain level, or included non-specific reads before statistical analyses.

To methodically tease apart the influence of environment and plant host on the rhizobiome composition, we analyzed beta dispersion to examine how community assembly strategies influenced the distance to the centroid between dry and wet local soil and rhizobiome samples, focusing on whether sample distribution was driven more by heterogeneric or convergent processes. We defined samples where beta dispersion distances close to 1 demonstrate increased dispersion, whereas smaller distances (e.g., 0.8) demonstrate more homogeneity (51). To complement our beta dispersion analysis, we calculated the Raup-Crick metric (RC_Bray_) based on Bray-Curtis dissimilarity for pairwise comparisons of samples within the same location. We defined samples where positive RC_Bray_ values (closer to 1) indicate communities are more dissimilar than expected by chance, negative values (closer to −1) indicate communities are more similar than expected by chance, and values near zero reflect greater stochasticity (47, 52, 53). Additionally, to assess phylogenetic differences within each location, we calculated beta Nearest Taxon Index (βNTI), which quantifies whether phylogenetic turnover between samples deviates from null expectations (54, 55). As established in prior literature, we interpreted |βNTI| > 2 as evidence for deterministic assembly, with positive values (>+2) indicating variable selection and negative values (<−2) indicating homogenous selection (52, 53). In contrast, |βNTI| ≤ 2 was interpreted as reflecting stochastic processes, consistent with ecological drift and/or dispersal (52, 53). For our effect size analysis, effect sizes for beta dispersion, RC_Bray_, and βNTI were evaluated using Hedges’ g, with absolute values of ~0.2, ~0.5, and ≥0.8 corresponding to small, moderate, and large differences between local soil and rhizobiome communities, respectively (56, 57).

To assess specific microbial families that contribute to stochasticity, we performed oligotyping analysis. For this analysis, we employed the QIIME 2 oligotype function (58) (v. 3.2) and the minimum entropy decomposition (MED) algorithm (59) to identify oligotypes within our aligned 16S rRNA amplicon sequences. We aligned our sequences using the MUSCLE function (60). To ensure an unbiased taxonomic assessment of oligotypes across various sample types, we set the number of components to 7 with a minimum substantive abundance parameter of 1 and included all features. These parameters were determined based on the entropy analysis of oligotype entropy peaks. We retained (LS: *n* = 2,248) and (RB: *n* = 2,876) oligotypes in this analysis.

In our taxonomic evaluation, we extracted specific features belonging to specific families from our previous taxonomic assessment. To evaluate whether host-mediated filtering is intensified under water-limited conditions, we performed indicator species analysis using the indicspecies (61) R package, applying the IndVal.g statistic with 999 permutations, and identified significant microbial families (and later genera) at *P* ≤ 0.001 across our cluster categories and sample types. From this analysis, we selected eight families (*Beijerinckiaceae*, *Chitinophagaceae*, *Micromonosporaceae*, *Pseudonocardiaceae*, *Rhizobiaceae*, *Rhizobiales Incertae Sedis*, *Saccharimonadaceae*, and *Steroidobacteraceae*), which contained specific members enriched in the dry rhizobiome (log_2_FC > 1), and are known for containing taxa with host-benefitting functions. We filtered features (*Beijerinckiaceae*: *n* = 590, *Chitinophagaceae*: *n* = 2,297, *Micromonosporaceae*: *n* = 781, *Pseudonocardiaceae*: *n* = 448, *Rhizobiaceae*: *n* = 339, *Rhizobiales Incertae Sedis*: *n* = 225, *Saccharimonadaceae*: *n* = 1,124, and *Steroidobacteraceae: n* = 242) that belonged to these families, prior to oligotyping analysis. We then set the number of components to 2 for the taxonomic analysis while maintaining a minimum substantive abundance parameter of 1. Following the taxonomic oligotyping analysis, families yielded the following total number of oligotypes: (*Beijerinckiaceae*: *n* = 15, *Chitinophagaceae*: *n* = 6, *Micromonosporaceae*: *n* = 11, *Pseudonocardiaceae*: *n* = 11, *Rhizobiaceae*: *n* = 12, *Rhizobiales Incertae Sedis*: *n* = 10, *Saccharimonadaceae*: *n* = 9, and *Steroidobacteraceae*: *n* = 12).

### Statistical analyses and data visualization

We used RStudio version 4.4.2 (62, 63) for all statistical analyses and data figure generation. We defined significance for all tests as having a *P* value or adjusted *P* value (multiple-testing correction applied as described below) less than 0.05.

For our studies of soil/climate properties, alpha diversity, community composition, assembly strategies, and relationships between these factors, we utilized the car (64), dplyr (65), devtools (66), dunn.test (67), pairwiseAdonis (68), tidyr (69), tidyverse (70), ggplot2 (71), FSA (72), broom (73), rstatix (74), readr (75), indicspecies (61), and vegan (76) packages.

We used one-way ANOVAs to detect differences among the factors including soil pH, phosphorus concentration (ppm P), potassium concentration (ppm K), carbon and nitrogen content (% total C and N), C:N ratio, soil texture (% sand, % silt, and % clay), and precipitation (rainfall) concerning the sample location and cluster category (dry, ambiguous, and wet). Additionally, we used Mantel tests to quantify associations between microbial community dissimilarity and environmental gradients across sample types (local soil vs rhizobiome) and cluster categories (wet, ambiguous, and dry). For each sample type and cluster category, we calculated Bray-Curtis dissimilarity matrices from amplicon community tables and Euclidean distance matrices for each environmental variable (77, 78). We subsequently assessed significance using permutation-based Mantel tests (9,999 permutations), with *P* values adjusted where appropriate using false discovery rate (FDR) correction (77, 78).

Furthermore, we analyzed the alpha diversity metrics across sample location, cluster, and pH/precipitation gradients using a one-way ANOVA to compare the means of the *S*_obs_, Faith’s PD, and H′ between local soil and rhizobiome samples. We performed a two-way ANOVA to analyze the relationship between our alpha diversity metrics, cluster categories, and pH/precipitation. When ANOVA identified differences among the factors, we performed Tukey HSD post hoc tests to determine where those differences were. Additionally, we applied the Kruskal-Wallis rank sum test to evaluate pairwise comparisons between sample locations and clusters for both the local soil and rhizobiome samples, analyzing our soil properties and precipitation (rainfall) data. When Kruskal-Wallis tests were significant, we performed post hoc Dunn tests with multiple-testing correction (Bonferroni) to identify pairwise differences.

In addition to ANOVA, we employed distance-based redundancy analysis (dbRDA) to evaluate the relationships between rainfall, soil properties, and microbial community composition. Using both parametric and non-parametric approaches allowed us to validate our findings across different statistical assumptions and ensured the robustness of our results. Finally, we conducted PERMANOVA analyses to determine whether bacterial community composition varied in relation to location, precipitation cluster category, soil properties, and precipitation. We then performed PERMANOVA pairwise comparisons to explore the pairs of groups individually between the factors described in our PERMANOVA analyses. In our analysis of how microbial diversity is impacted by climate and soil properties, we performed both *t*-test and linear regression analyses to further explore these relationships.

To assess differences in the variability of community composition within each cluster category and evaluate category-specific dispersion patterns, we conducted analyses of multivariate homogeneity of group dispersions (employing the betadisper function). We assessed the differences between groups based on the mean distance to the centroid for each category. This evaluation helped us determine the probability of overlap between the cluster categories. Within our betadisper test, we performed a permutation test and a pairwise permutation-based test to examine sample heterogeneity within each cluster category. Finally, we used Pearson’s Chi-squared tests to find statistical differences in ASV composition between sample types (LS and RB) and the cluster categories.

We used beta dispersion distances and the beta-group-significance function in QIIME 2 to infer community variability within a location and between sample types. We examined the differences between the LS and RB beta dispersion distances within a location by employing a one-way ANOVA. To provide evidence for deterministic versus stochastic community assembly, we calculated the RC_Bray_ metric by comparing the observed Bray-Curtis dissimilarity between each pair of samples to a null distribution generated from 999 randomizations of the community matrix (47, 52, 53). In each randomization, we shuffled species abundances across samples (within each species) while preserving the total abundance of each species across all samples (47, 52, 53). We then calculated the RC_Bray_ value as the standardized difference (z-score) between the observed and mean null dissimilarity (47, 52, 53). Our normalized z-scores ranged from −1 to 1 (47, 52, 53). We then used Kruskal-Wallis tests to evaluate differences in RC_Bray_ distances across groups. When significant, we subsequently performed post-hoc Dunn tests with Bonferroni correction to identify pairwise differences.

To provide evidence for host-selection in drought-associated rhizobiomes, we calculated phylogenetic community turnover using βNTI, which was calculated from QIIME 2, using our previously generated exported feature table and rooted phylogenetic tree. We then computed the abundance-weighted, symmetric β mean nearest taxon distance (βMNTD) among shared ASVs of all sample pairs using the same patristic distances derived from the phylogeny (54, 55). To generate the null expectation, we performed 999 taxa-label randomizations of the phylogenetic tree and recalculated βMNTD for each permutation (54, 55). Finally, the βNTI values were calculated as the standard effect size of the observed βMNTD relative to the null distribution (54, 55). To evaluate differences in βNTI distances across groups, we used Kruskal-Wallis tests, with post-hoc Dunn tests with Bonferroni correction, as described previously.

Finally, we quantified within-location effect sizes for beta dispersion, RC_Bray_, and βNTI between local soil and rhizobiome communities using Hedges’ *g*, where positive values indicate greater distances in local soil and negative values indicate greater distances in the rhizobiome. For the oligotyping analysis, we employed the beta dispersion tests previously described, along with *t*-tests for family abundance level differences.

We utilized the RStudio packages ggplot2 (71), patchwork (79), dplyr (65), multcomp (80), multcompView (81), agricolae (82), tidyr (69), venn (83), tidyverse (70), vegan (76), cluster (84), sf (85), maps (86), reshape2 (87), pheatmap (88), RColorBrewer (89), car (64), gridExtra (90), phyloseq (91), igraph (92), ggnewscale (93), readr (75), ggthemes (94), ggtext (95), ggVennDiagram (96), randomcoloR (97), stringr (98), indicspecies (61), and VennDiagram (99) to visualize the analyses performed in this study.

## RESULTS AND DISCUSSION

We processed a total of 13,087,481 (local soil) and 16,677,859 (rhizobiome) raw reads. After quality control, we retained 11,759,436 reads (2,462 ASVs) for the local soil and 14,627,083 reads (3,039 ASVs) for the rhizobiome (Table S2). In the local soil samples, 92% (2,267 ASVs) of the ASVs resolved to the genus level, and 64% (1,567 ASVs) resolved to the species level. We observed similar resolution across the rhizobiome samples, where 93% (2,815 ASVs) resolved to the genus and 65% (1,985 ASVs) resolved to the species level. The five most abundant phyla in the local soil samples were Proteobacteria (22%, 532 ASVs), Actinobacteriota (11%, 278 ASVs), Bacteroidota (9.8%, 242 ASVs), Chloroflexi (8.1%, 200 ASVs), and Acidobacteriota (7.6%, 187 ASVs) (Table S2). We observed similar dominant phyla across the rhizobiome samples, with Proteobacteria (23%, 691 ASVs) being the most dominant, followed by Actinobacteriota (10%, 315 ASVs), Bacteroidota (9.9%, 302 ASVs), Planctomycetota (7.5%, 229 ASVs), and Chloroflexi (7.5%, 228 ASVs) (Table S2). Of all our samples, 0.12% (3 ASVs) from the local soil and 0.13% (4 ASVs) from the rhizobiome were unclassified.

### Regional precipitation shaped both local soil and plant-associated microbial communities

**Fig 1:**
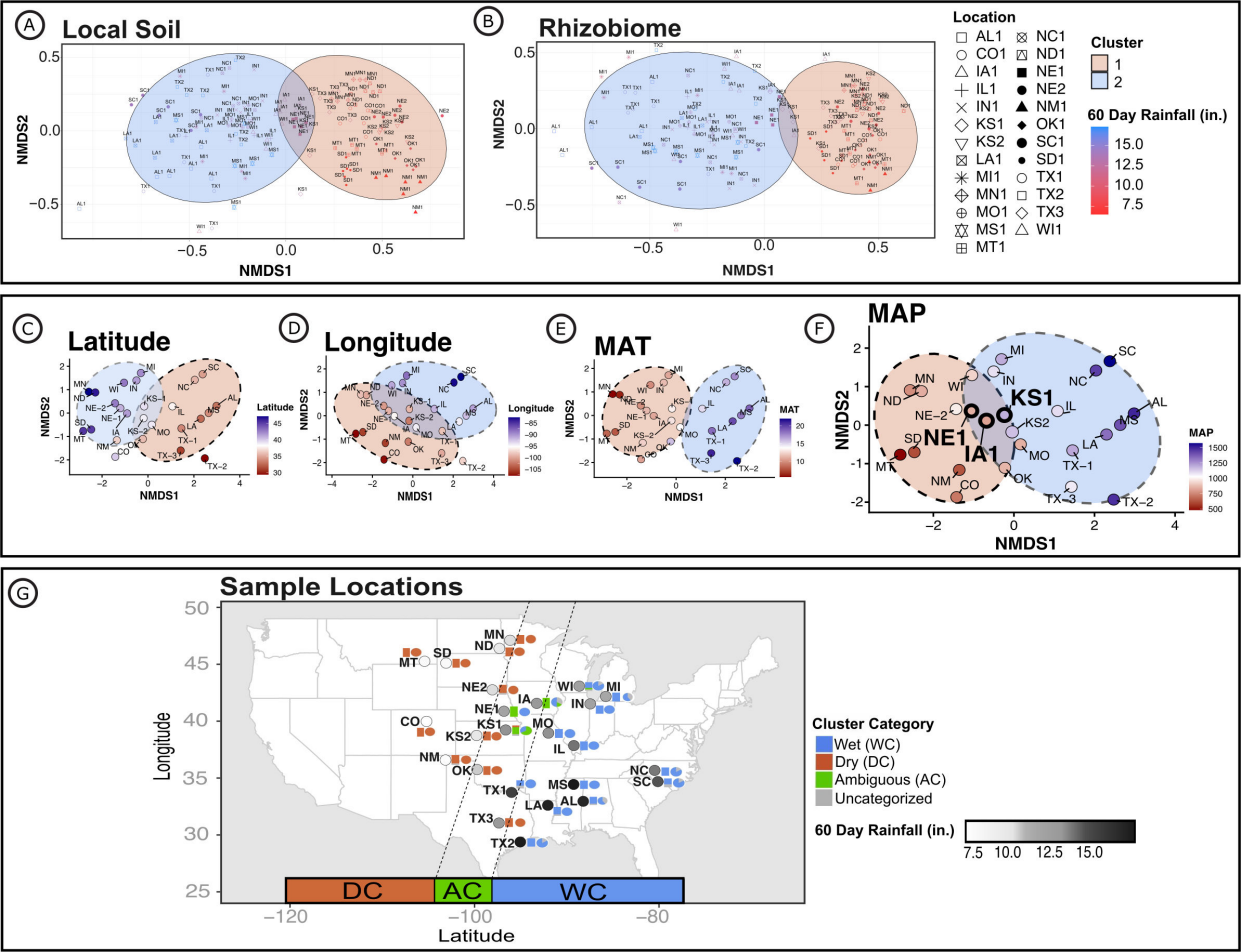
Precipitation-associated sample clustering and geospatial analysis. (**A**) NMDS clustering of local soil samples with k-means clustering and optimized silhouette scores showing distinct groupings with 95% confidence ellipses. Each point represents a sample, colored according to NOAA’s 60-day maximum precipitation data at the collection site. (**B**) NMDS clustering of rhizobiome samples with the same clustering criteria. Sample points are uniquely marked and colored by precipitation (rainfall), consistent with panel A. (**C–F**) NMDS clustering of location specific geographic (latitude and longitude) and climate properties (mean annual temperature [MAT] and mean annual precipitation [MAP]), with the same clustering criteria as described in panel **A**. (**G**) Map of biogeographical survey sites, with markers colored by NOAA’s 60-day maximum precipitation (circle) values. Stacked bar graphs within the map display the proportion of local soil samples assigned to each NMDS-defined group at each site. The adjacent pie charts summarize group assignments for rhizobiome samples.

We applied non-metric multidimensional scaling (NMDS) and k-means clustering, using optimal silhouette scores, and identified two distinct groups in both the local soil (silhouette score = 0.21) and rhizobiome (silhouette score = 0.22) sample sets (Fig. 1A and B). Because these clusters closely aligned with regional precipitation patterns, we denoted them as “dry-categorized” and “wet-categorized.” Samples falling within the transitional zone between these two primary clusters were designated as “ambiguously categorized.” Each location was then assigned to the category to which the majority of its samples fell.

Using this classification scheme, 10 samples were designated as dry-categorized local soil (DC_LS_): MT1, SD1, ND1, MN1, CO1, KS2, NM1, NE2, OK1, and TX3. Three samples (KS1, NE1, and IA1) were classified as ambiguously categorized local soil (AC_LS_), while 12 samples (WI1, MI1, IN1, MO1, IL1, TX1, TX2, LA1, AL1, NC1, and SC1) were organized as wet-categorized local soil (WC_LS_). Assignments for the dry-categorized local soil samples matched those of the dry-categorized rhizobiome (DC_RB_). However, the other sample assignments exhibited slight changes from the local soil for the ambiguously (AC_RB_) and wet (WC_RB_) categorized rhizobiomes, with IA1 and NE1 reclassified into the WC_RB_ category.

We surmise that the dry-categorized samples showed more consistent assignments than wet- or ambiguous-categorized samples, consistent with long-standing associations between the dry variety of *A. gerardii* and its rhizobiome (6, 15, 22, 100). In contrast, wet and ambiguous samples may be less tightly linked to host identity, possibly due to shifting historical precipitation boundaries and increasing aridity in previously moist regions (27, 101, 102).

To determine whether the observed sample clustering reflected underlying environmental structure rather than microbial composition alone, we conducted an independent unsupervised clustering analysis using site-level climate variables from ClimateNA (v.7.60) (103) (Fig. 1C–F; Table S3). We found that clusters differed significantly in longitude, latitude, mean annual precipitation (MAP), and mean annual temperature (MAT) in both local soil and rhizobiome samples. In soil communities, the strongest differences were observed for longitude and MAP (Kruskal-Wallis: p_adj_ ≤1.0 × 10⁻⁶), followed by latitude and MAT (p_adj_ = 6.4 × 10⁻⁵). Additionally, clusters differed significantly in longitude (p_adj_ = 4.7 × 10⁻⁶), MAP (p_adj_ = 3.0 × 10⁻⁵), and both latitude and MAT (p_adj_ = 5.8 × 10⁻⁴). Notably, each environmental variable consistently resolved into two major location-based clusters that closely mirrored the microbial community patterns. We therefore used these previously defined clustering classifications as precipitation-based, *a priori* supported groups in all subsequent analyses.

To further assess the impact of regional precipitation on local soil and rhizobiome composition, we used the NOAA (34) (v. 2) rainfall database to calculate maximum 60-day precipitation (relative to annual rainfall at each 2023 sampling location) and mapped cluster distributions across the precipitation gradient (Fig. 1G). Notably, we observed a clear separation between the dry and wet categories along the 100^th^ meridian, the North American “arid-humid divide,” with the ambiguous samples comprising the mesic transition zone. This clustering suggests that regional precipitation strongly shapes microbiome composition across geographically diverse sites (104), potentially outweighing local climate, geographic, and soil property differences (105, 106).

Consistent with this pattern, one-way ANOVA confirmed significant differences in rainfall depth among cluster categories across sample types (*P* < 2.2 × 10^−16^ for both). Additionally, pairwise Kruskal-Wallis post hoc tests showed the strongest differences between dry and wet categories in local soil (p_adj_ = 3.92 × 10⁻²⁰) and rhizobiome samples (p_adj_ = 2.08 × 10⁻⁹), whereas dry-ambiguous comparisons were weaker and wet-ambiguous comparisons were not significant or marginal (Table S3).

We further applied PERMANOVA and distance-based redundancy analysis (dbRDA) and found that rainfall significantly influenced both local soil and rhizobiome community structure: (PERMANOVA: *P* = 0.001). In local soil, dbRDA explained 59.1% of variance by individual location (F = 7.51, *P* = 0.001) and 27.1% by cluster category (F = 29.78, *P* = 0.001). Similarly, in the rhizobiome, location explained 55.6% of variance (F = 6.22, *P* = 0.001) and cluster category explained 21.9% (F = 19.8, *P* = 0.001). However, pairwise PERMANOVA revealed significant differences among all local soil categories (p_adj_ = 0.003), whereas rhizobiome comparisons involving the ambiguous group were not significant (WC_RB_ vs AC_RB_: p_adj_ = 0.16; DC_RB_ vs AC_RB_: p_adj_ = 0.07). Although we acknowledge that sampling at a single summer time point does not capture seasonal microbial variation and that our precipitation data from NOAA and ClimateNA annual averages do not account for all climatic drivers of community composition (107–109), our results support previous findings (23, 110) in which regional precipitation influences local soil communities more than plant host-associated rhizobiome communities. Here, we speculate that under adverse hydrologic conditions, the plant host plays an increasingly important role in shaping and regulating the structure of its associated rhizobiome community (1, 111, 112). Next, we wanted to ask if and to what extent physicochemical soil properties contributed to this variation.

### Soil physicochemical properties and bacterial diversity were primarily influenced by geographic location rather than regional precipitation

We observed that soil physicochemical properties (Table S3) aligned more strongly with individual sampling locations than with our *a priori* defined precipitation-based cluster categories. Our results suggest that site-level soil variation may shape environmental patterns beyond precipitation alone. Although we recognize that the local soil may not fully capture the root microenvironment (113), it still provides a useful baseline of site-level conditions influencing the rhizosphere. This is supported by our finding that local soil and rhizosphere communities were largely not significantly different across sites, suggesting that local soil properties reasonably reflect conditions shaping both sample types (112, 114).

All soil properties differed significantly among locations, reflecting pronounced spatial heterogeneity. In contrast, only soil pH (ANOVA: *P* = 1.05 × 10^−12^), potassium content (ANOVA: *P* = 1.05 × 10^−12^), sand content (ANOVA: *P* = 1.02 × 10^−2^, and silt content (ANOVA: *P* = 1.90 × 10^−4^) differed significantly across our precipitation-based cluster categories. These same variables were also significantly associated with local soil community structure by dbRDA (*P* = 0.001), each explaining ~25% of the variance (25.0%–25.5%). We speculate that this likely reflects high local variability masking broader regional trends (115). Given these patterns, we next asked whether differences in microbial community composition were systematically related to environmental gradients across sites.

**Fig 2:**
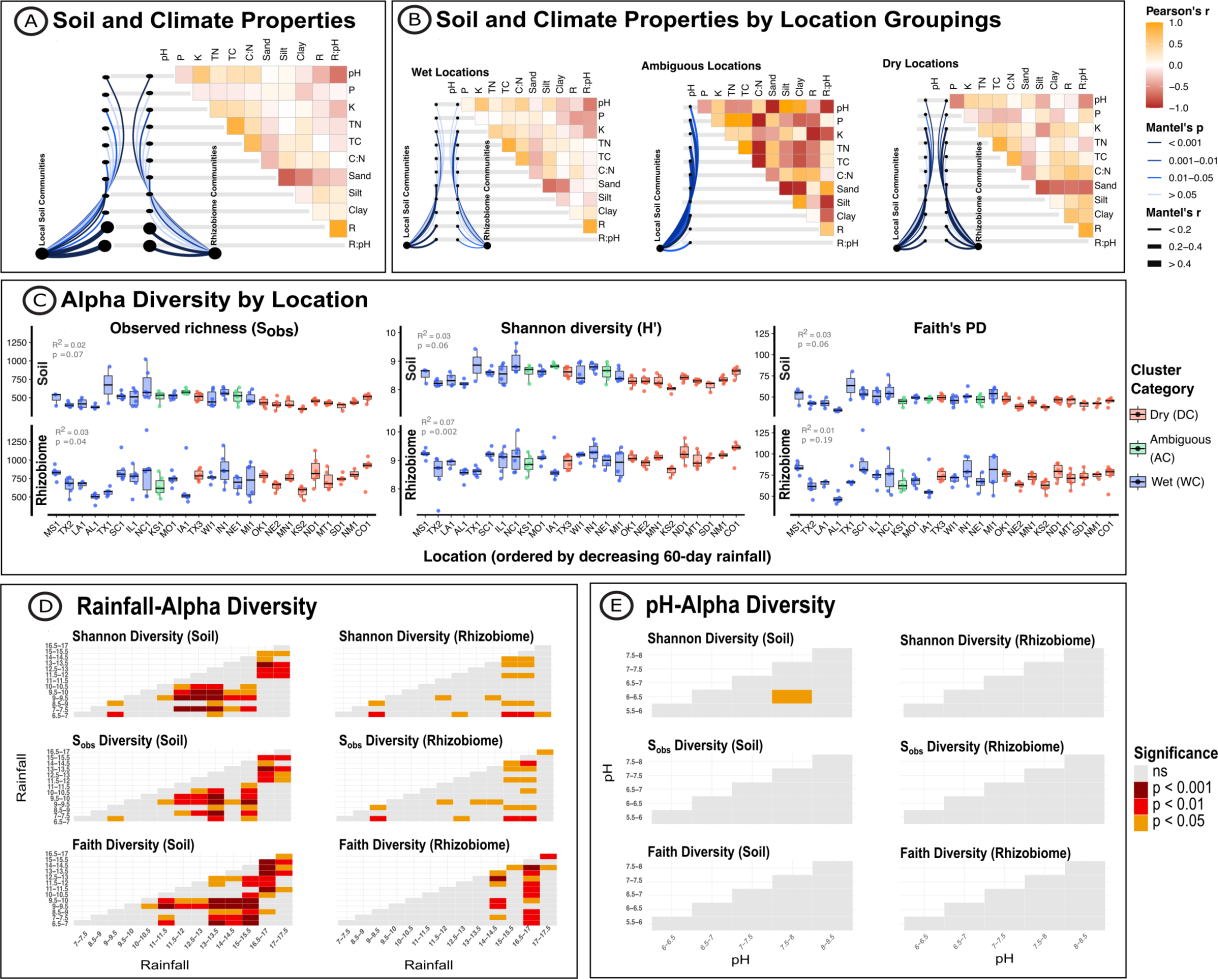
Cluster soil physicochemical properties and alpha diversity across cluster categories and soil property gradients. (**A**) Pearson correlation heatmap (right) showing relationships among soil physicochemical properties (pH, P, K, N, C, C:N, sand, silt, clay), precipitation (R, rainfall), and rainfall pH ratio (R:PH) for local soil and rhizobiome samples. Colors indicate the strength and direction of Pearson’s *r* (orange = positive, red = negative). Mantel tests (left) quantify associations between environmental distance matrices and microbial community dissimilarity for local soil and rhizobiome communities. Line color denotes Mantel *P* value significance (<0.001, 0.001–0.01, 0.01–0.05, >0.05), while line thickness reflects Mantel *r* strength (<0.2, 0.2–0.4, >0.4). (**B**) The same analyses shown in panel A are presented separately for wet, ambiguous, and dry cluster categories. Panels illustrate how relationships between soil physicochemical properties, precipitation, and microbial community structure differ across precipitation-defined environments for both local soil and rhizobiome communities. (**C**) Linear regression analysis was conducted to evaluate the relationship between precipitation and alpha diversity metrics, including *S*_obs_, H′, and Faith’s PD, in local soil (top panels) and rhizobiome (bottom panels) communities. Samples were arranged along a gradient of decreasing precipitation and visualized using violin plots. The plot color denotes the location’s cluster category (dry, ambiguous, and wet). Displayed in each of the plots are the *P* and *R*^2^ values from the regression analysis. (**D**) Comparison of alpha diversity metrics between local soil and rhizobiome samples across environmental precipitation (rainfall) categories (all samples used) (assessed using independent *t*-tests. Statistical significance is indicated as follows: ns (gray) = *P* > 0.05, * (orange) *P* < 0.05, ** (red) *P* < 0.01, *** (brown) *P* < 0.001. (**E**) Comparison of alpha diversity metrics between local soil and rhizobiome samples across pH categories (all samples used), also assessed in the same manner as in panel C. Statistical significance is indicated as follows: ns (gray) = *P* > 0.05, * (orange) *P* < 0.05, ** (red) *P* < 0.01, *** (brown) *P* < 0.001.

Mantel tests revealed that environmental gradients were significantly associated with microbial community dissimilarity across both the local soil and rhizobiome, although drivers differed between sample types (Fig. 2A; Table S3). However, consistent with our previous findings, precipitation-related metrics were the dominant correlates of community turnover, with rainfall (R) and the rainfall–pH ratio (R:pH) showing the strongest associations. While both pH and precipitation were influential, their direct vs indirect effects remain difficult to disentangle (116, 117).

In the local soil, these precipitation-based variables exhibited the largest effect sizes (R: *r* = 0.56, *P* < 0.001; R: pH: *r* = 0.51, *P* < 0.001), highlighting strong climatic control. These patterns align with pedogenic processes in which rainfall drives leaching of basic cations, such as potassium, which can lower soil pH (118). Other soil properties were also significant but weaker, including pH (*r* = 0.17), sand (*r* = 0.19), silt (*r* = 0.15), clay (*r* = 0.06), and C:N ratio (*r* = 0.09; all *P* ≤ 0.044). In the rhizobiome, rainfall metrics remained significant but were reduced relative to local soil (R: *r* = 0.36, *P* < 0.001; R:pH: *r* = 0.37, *P* < 0.001). Among our other variables, only pH (*r* = 0.12, *P* < 0.001), potassium (*r* = 0.13, *P* = 0.0012), and sand content (*r* = 0.09, *P* = 0.0068) were significantly tied to rhizobiome community turnover, whereas carbon, nitrogen, phosphorus, and other soil variables were insignificant. This suggests that rhizobiome communities are more strongly shaped by host-mediated filtering than bulk soil conditions.

We next asked whether environmental drivers of microbial turnover differed across sample types (dry vs wet) (Fig. 2B; Table S3). In dry rhizobiomes, community turnover remained strongly coupled to environmental gradients, with significant associations for potassium (*r* = 0.25), silt (*r* = 0.26), rainfall (*r* = 0.23), and R:pH (*r* = 0.30; all *P* < 0.001). Additionally, wet rhizobiomes showed fewer and weaker significant relationships, limited to potassium, sand, rainfall, and R:pH (*r* = 0.09–0.24; all *P* < 0.018), suggesting reduced environmental sensitivity under hydrologically favorable conditions (119).

Dry local soils showed an even stronger parallel response, with significant associations for potassium (*r* = 0.32), sand (*r* = 0.37), silt (*r* = 0.36), rainfall (*r* = 0.30), and R:pH (*r* = 0.35; all *P* < 0.001). While our wet local soils retained precipitation effects, they were at lower magnitudes (R: *r* = 0.30; R:pH: *r* = 0.21; all *P* < 0.001). Taken all together, we interpret this as amplified filtering under water limitation.

Strikingly, ambiguous local soils showed a shift in dominant controls on community structure. Here, we discovered that edaphic properties, rather than precipitation, were the strongest correlates of dissimilarity, including phosphorus (*r* = 0.42), total nitrogen (*r* = 0.43), total carbon (*r* = 0.43), C:N ratio (*r* = 0.42), and clay (*r* = 0.44; all *P* ≤ 0.0032). Notably, precipitation was no longer significant (*r* = 0.08, *P* = 0.19), suggesting a transition from climate-dominated to soil property-driven microbial assembly in these intermediate environments (120–122). Given this pronounced role of local edaphic filtering, we next assessed whether alpha diversity similarly reflects shifts in community organization across our precipitation categories.

We observed that, across the 25 locations, all alpha diversity metrics (Table S3) were significantly tied to location (ANOVA: LS: *S*_obs_ *P* = 1.06 × 10^−8^, Faith’s PD *P* = 4.91 × 10^−10^, and H′ *P* = 7.38 × 10^−10^), and (RB: *S*_obs_ *P* = 6.56 × 10^−6^, Faith *P* = 4.54 × 10^−8^, and H′ *P* = 1.98 × 10^−5^; Fig. 2C, top panels). On the other hand, while local soil alpha diversity metrics significantly differed across the local soil cluster categories (ANOVA: LS: *S*_obs_ *P* = 6.02 × 10^−5^, Faith’s PD = 1.55 × 10^−3^, and H′ *P* = 8.75 × 10^−6^), we found no significant differences between our rhizobiome cluster categories, possibly due to shielding from the host (Table S3).

Guided by our Mantel test results, we explored the influence of pH and precipitation on microbial diversity. Although pH and precipitation strongly structure microbial communities, their direct effects are difficult to separate from co-varying environmental factors. However, well-established links between these variables and alpha diversity motivated further analysis (104, 105, 107, 117, 123) (Fig. S1). We found that rainfall significantly affected all alpha diversity metrics across ANOVA: LS and RB (*S*_obs_ *P* = 0.05, Faith’s PD *P* = 3.82 × 10^−3^, H′ *P* = 7.17 × 10^−3^) and between the sample types (LS vs RB) (*P* < 2 × 10^−16^). Similar results were observed in our linear regression analysis comparing the alpha diversity metrics of rhizobiomes ordered by varying levels of rainfall (Fig. 2C, bottom panels).

Pairwise tests further revealed that the most dramatic differences in diversity were observed between the following precipitation categories (60-day maximum rainfall, 0.5 inch bins) across sample types: H′: LS: p_adj_ = 2.21 × 10^−3^ (9 to 9.5 vs 12.5 to 13), RB: p_adj_ = 3.21 × 10^−3^ (6.5 to 7 vs 15 to 15.5); *S*_obs_: LS: p_adj_ = 2.05 × 10^−3^ (9 to 9.5 vs 12.5 to 13), RB: p_adj_ = 3.99 × 10^−3^ (6.5 to 7 vs 16.5 to 17); and Faith’s PD: LS: p_adj_ = 2.21 × 10^−3^ (13 to 13.5 vs 16.5 to 17); RB: p_adj_ = 6.05 × 10^−4^ (14 to 14.5 vs 16.5 to 17) (Fig. 2D; Table S3). In this analysis, the local soil showed the strongest diversity shifts, particularly among moderate precipitation categories, whereas the rhizobiome exhibited more variable changes mainly at precipitation extremes. This suggests that soil communities respond more dynamically to subtle rainfall variations within the “ambiguous” transition zone (124), while rhizobiomes remain buffered except under extreme precipitation differences (125).

Linear regression further revealed significant changes of relative abundance across the precipitation gradient for seven phyla: *Crenarchaeota*, *Desulfobacterota*, *MBNT15*, *Planctomycetota*, *RCP2-54*, *Verrucomicrobiota*, and *WPS-2* with *P* values of *P* = 4.00 × 10^−2^, *P* = 1.00 × 10^−2^, *P* = 9.41 × 10^−3^, *P* = 2.11 × 10^−2^, *P* = 1.09 × 10^−2^, *P* = 3.46 × 10^−4^, and *P* = 9.64 × 10^−4^ respectively (Fig. S2). Notably, all phyla except *Crenarchaeota* (slope = −1.22 × 10^−4^; R^2^ = 0.306) increased in relative abundance with greater precipitation. We hypothesize that the decrease in *Crenarchaeota* might be due to their roles as ammonia-oxidizing archaea (AOA), which might not be in high demand or feasible under waterlogged conditions (126). In contrast, no significant changes in alpha diversity (ANOVA: *S*_obs_ *P* = 0.73, Faith’s PD = 0.59, H′ *P* = 0.49) or bacterial relative abundance were found across the pH gradient (categories were separated by a 0.5 change in pH), with differences only observed between sample types (*P* < 2 × 10^−16^) and cluster category for Faith’s PD (*P* = 2.08 × 10^−3^) (Fig. 2E). Additionally, two-way ANOVA identified a significant interaction between soil pH and cluster category on Shannon diversity (*P* = 1.54 × 10⁻²). To further examine this relationship, we performed linear regression analyses comparing alpha diversity in rhizobiome and local soil samples across locations, ordered by decreasing pH (Fig. S3).

Pairwise analysis showed no significant differences in rhizobiome alpha diversity and only minimal differences across soil pH categories, suggesting compositional shifts without major changes in richness (Table S3). This minimal variation across our pH subdivisions supports host legacy effects (10, 13, 127). This pattern is consistent with our previous reciprocal garden experiment (22), in which wet host varieties exhibited reduced richness (*S*_obs_) when planted in dry site soils. Building on our findings that precipitation drives rhizobiome differences under abiotic extremes, we next explored how precipitation, sample type, and cluster category shape ASV distributions.

### Prokaryotic community composition revealed patterns that differentiated our cluster categories

**Fig 3:**
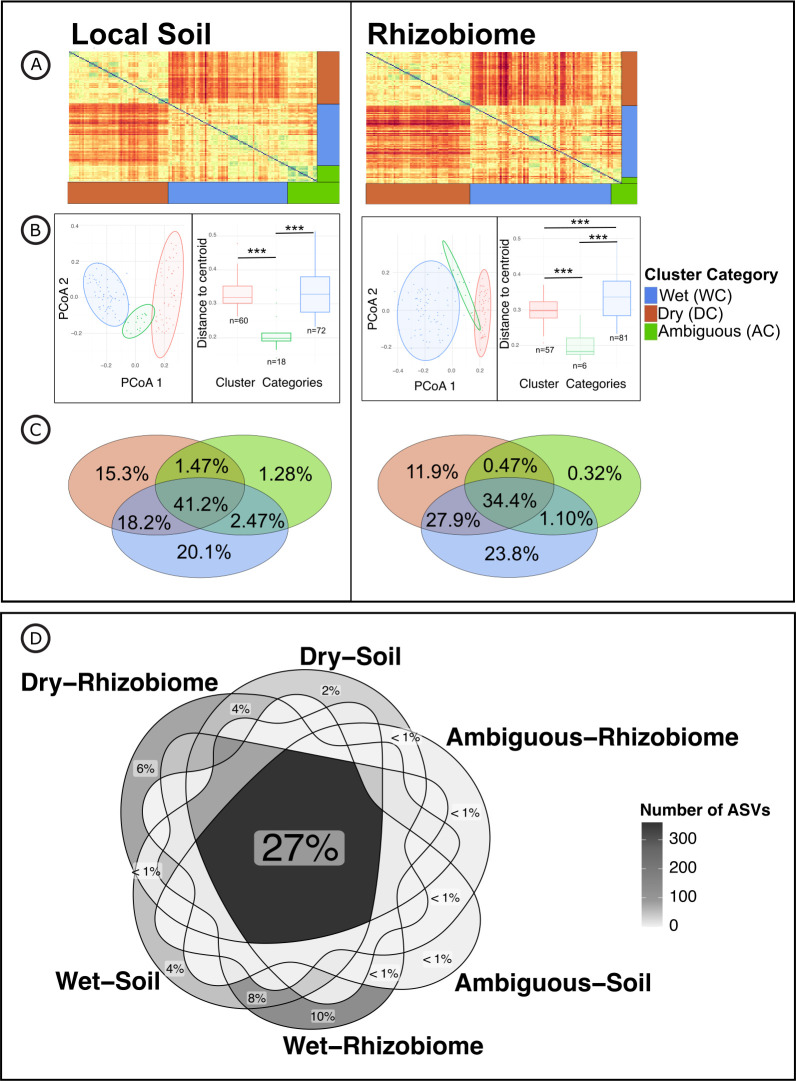
Beta diversity patterns and ASV distribution across cluster categories and sample types. (**A**) Bray-Curtis dissimilarity heatmaps illustrating large-scale community composition trends in (**A**) local soil and (**B**) rhizobiome samples, clustered by their cluster assignments (dry, ambiguous, and wet). (**B**) PCoA plots of beta dispersion, showing community variability within cluster category (dry, ambiguous, and wet) for each sample type. The accompanying box plots display the distance of each sample to the group centroid. Statistical significance is defined as follows: ns = *P* > 0.05, *P < 0.05, **P < 0.01, ***P < 0.001. (**C**) Venn diagrams depicting the distribution of shared and unique ASVs across dry, ambiguous, and wet cluster categories for local soil and rhizobiome samples. (**D**) Combined Venn diagram comparing ASV overlap between local soil and rhizobiome sample types across dry and wet cluster categories. Only the main sections have been labeled with percentages.

We observed that local soil and rhizobiome samples showed similar microbial community composition trends across our cluster categories (Fig. 3A). Interestingly, we observed that the dry-categorized samples were more similar to each other than wet-categorized samples, suggesting that increased abiotic stress may impose stronger filtering on microbial community structure (128, 129). In line with this observation, we found that beta diversity dispersion differed significantly among cluster categories for both local soil and rhizobiome communities (PERMANOVA, *P* = 0.001; F-statistic LS: 28.4, RB: 24.9), with BetaDisper distances to the median showing variable within-group dispersion (DC_LS_ = 0.33, DC_RB_ = 0.30; AC_LS_ = 0.21, AC_RB_ = 0.20; WC_LS_ = 0.33, WC_RB_ = 0.34). Furthermore, pairwise comparisons were significant across most categories (DC_LS_ vs AC_LS_: *P* = 1.57 × 10^−13^, AC_LS_ vs WC_LS_: *P* = 1.35 × 10^−9^; DC_RB_ vs AC_RB_: *P* = 1.75 × 10^−7^, AC_RB_ vs WC_RB_: *P* = 4.79 × 10^−6^, and DC_RB_ vs WC_RB_: *P* = 1.00 × 10^−3^), except DC_LS_ vs WC_LS_ (*P* = 0.91). These results support that microbial communities vary within clusters, with greater heterogeneity in the dry and wet categories, and comparatively lower dispersion in the ambiguous transition group (22).

Although dry and wet clusters did not differ significantly in ASV composition across sample types, two observations prompted us to perform further analysis: (i) dry rhizobiome samples were more similar to each other than dry soils, and (ii) dry and wet rhizobiomes differed strongly (Fig. 3B). To further resolve these patterns, we quantified shared and unique ASVs among dry, ambiguous, and wet cluster categories using overlap analysis. We found that shared ASVs across all cluster categories (41.2% LS and 34.4% RB), accounted for the largest proportion, while wet clusters had more unique ASVs (20.1% LS and 23.8% RB), followed by the dry-categorized cluster (15.3% LS and 11.9% RB) (Fig. 3C). Across sample types (LS and RB), we observed that 26.5% of ASVs were shared among all categories (Fig. 3D; Table S4). Consistently, we observed that wet clusters contained more unique ASVs than dry clusters, which may reflect microbial legacy effects and higher diversity due to favorable abiotic conditions (130, 131). In contrast, we hypothesize that the dry-categorized clusters may favor specialists over generalists, reducing ASV diversity (132, 133). Correspondingly, the limited overlap of ASVs between clusters and sample types likely reflects phylogenetic divergence and niche specialization, where microbes adapted to specific precipitation pressures may not suit contrasting environments (134).

We confirmed significant differences in unique ASVs across cluster categories (Chi-squared, *P* = 2.2 × 10^−16^), and between wet and dry categories (*P* = 0.05), but not sample type (*P* = 0.557). Our results suggest that complex environmental and climate factors have a more profound impact on shaping the number of unique ASVs than the localized effects of individual plant hosts.

### Deterministic processes exerted a stronger influence on community composition in the rhizobiome than in the local soil across the most arid locations

**Fig 4:**
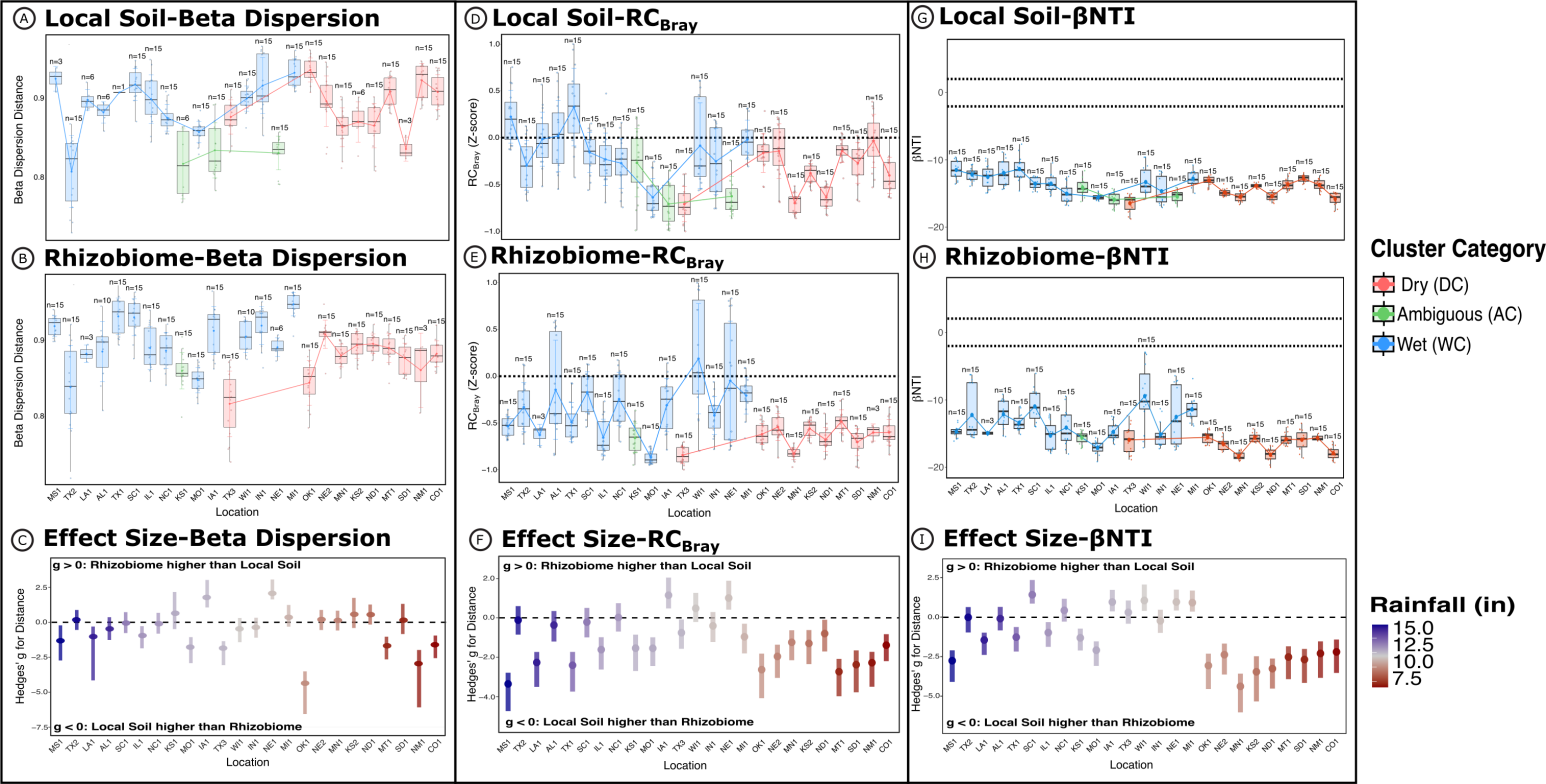
Within-site community variability across local soil and rhizobiome samples. (**A**) Beta dispersion analysis of local soil samples, assessing within-site variability across locations. Sample points are colored by their cluster category, with lines representing the mean distance to the centroid for each category and shaded bars indicating standard deviation. Sites are arranged in order of decreasing precipitation. (**B**) Rhizobiome beta dispersion across the same locations and cluster categories. (**C**) Effect size (Hedges’ *g*) for beta dispersion, quantifying within-location differences between rhizobiome and local soil communities. Positive values indicate greater dispersion in the rhizobiome, whereas negative values indicate greater dispersion in local soil. Error bars represent 95% confidence intervals, and the dashed line denotes no difference (*g* = 0). (**D**) Raup-Crick Bray (RC_Bray_) analysis of local soil samples, assessing within-site variability across locations. Sites are arranged in order of decreasing precipitation. (**E**) Rhizobiome RC_Bray_ values across the same locations and cluster categories. (**F**) Effect size (Hedges’ *g*) for RC_Bray_, where positive values indicate stronger deviation from null expectations in the rhizobiome relative to local soil. (**G**) Local soil βNTI values, representing phylogenetic turnover relative to null models, with values beyond ± 2 indicating deterministic selection. (**H**) Rhizobiome βNTI values**,** showing enhanced phylogenetic structuring under specific precipitation contexts. (**I**) Effect size (Hedges’ *g*) for βNTI**,** summarizing compartment-level differences in deterministic phylogenetic assembly. Positive values indicate stronger deterministic structuring in the rhizobiome.

Building on these observations of increased community convergence under dry conditions, we next assessed patterns of community variability and assembly. Both local soil and rhizobiome samples showed high beta-dispersion distances across dry and wet locations and cluster categories, which is common among grassland microbiomes (135) (Fig. 4A and B; Table S5). However, we observed pronounced differences in within-habitat variability in the dry-categorized sites. Specifically, in eight of 10 dry locations, beta dispersion differed significantly between local soil and rhizobiome communities (ANOVA: Dry: TX3: *P* = 3.43 × 10^−5^; NE1: *P* = 5.41 × 10^−6^; OK1: *P* = 1.45 × 10^−11^; KS2: *P* = 3.08 × 10^−2^; ND1: *P* = 1.20 × 10^−3^; MT1: *P* = 6.02 × 10^−3^; SD1: *P* = 5.06 × 10^−3^; NM1: *P* = 1.52 × 10^−3^; and CO1: *P* = 8.69 × 10^−4^), whereas only one wet comparison was significant (ANOVA Wet: MI1: *P* = 3.02 × 10^−2^). Notably, in the two driest locations, CO1 and NM1, local soils exhibited significantly greater beta dispersion than their corresponding rhizobiome communities, indicating higher compositional heterogeneity in the soil communities, a trend largely unique to the dry sites (Fig. 4C).

With this trend in mind, we next asked whether these observed differences in community composition among samples within a location reflected shifts in dominant assembly mechanisms rather than differences in overall community heterogeneity. To address this, we used RC_Bray_ analysis (Fig. 4D and E; Table S5) and showed significant differences in assembly processes between dry and wet cluster categories and sample types (Kruskal-Wallis: χ² = 166.1, df = 3, *P* < 9.0 × 10^−36^).

Additionally, the most dramatic contrasts in the RC_Bray_ metric were between dry rhizobiome (DC_RB_) and all other groups (Kruskal-Wallis post hoc pairwise tests: WC_LS_: p_adj_ = 5.079 × 10^−34^; WC_RB_: p_adj_ = 1.02 × 10^−14^; and DC_LS_: p _adj_ = 2.34 × 10^−15^), highlighting a fundamental distinct assembly regime of the DC_RB_ community (Fig. 4F). These results indicated that rhizobiome communities in the driest locations exhibited greater deviation from null expectations, consistent with intensified deterministic structuring (136). We therefore interpreted these patterns as evidence that, under extreme water limitation, the relative influence of host-mediated selection and abiotic filtering increased, constraining community membership and reducing the role of random dispersal and drift (51, 119, 137). In this context, dry cluster rhizobiomes might represent a selectively filtered subset of the regional species pool, leading to a more predictably assembled and structurally stable community compared to the surrounding local soils (138).

To directly test whether these patterns reflected deterministic selection rather than stochastic assembly, we quantified phylogenetic turnover using βNTI (Fig. 4G and H). In line with our previous results, across all locations, βNTI differed strongly between sample types, with root communities exhibiting significantly different phylogenetic turnover than soils (Kruskal-Wallis: χ² = 45.0, df = 2, *P* = 1.92 × 10⁻¹¹). Stratification by cluster category further structured βNTI patterns within both compartments (LS: χ² = 61.4, df = 2, *P* = 4.72 × 10⁻¹⁴; RB: χ² = 109, df = 2, *P* = 1.84 × 10⁻²⁴), with all pairwise contrasts among dry, ambiguous, and wet cluster categories remaining significant after correction (p_adj_ = 3.6 × 10⁻³). Critically, compartmental differences were not uniform across our cluster categories. In dry locations, βNTI differed markedly between local soil and rhizobiome communities (χ² = 101, df = 1, *P* = 1.11 × 10⁻²³), whereas no significant soil-rhizobiome differences were observed in ambiguous locations (*P* = 0.27), and only weak differences were detected in wet sites (*P* = 0.054) (Fig. 4I; Table S5). Critically, the effect size analysis revealed a clear trend along the precipitation gradient, with soil-rhizobiome βNTI differences becoming progressively stronger toward drier sites. Based on these results, we hypothesized that in the driest environments, the plant acted as a stronger ecological filter, favoring stress-tolerant and host-compatible taxa and thereby driving the pronounced deterministic patterns we observed exclusively in the dry rhizobiome (139).

Together, our results suggested that drought fundamentally shifted rhizobiome assembly from largely stochastic dynamics toward stronger, deterministic processes. In the rhizobiomes of the driest environments, both RC_Bray_ and βNTI indicated a synergistic interplay between host-mediated selection and environmental stress, whereby intensified host filtering and abiotic constraints favored a restricted subset of microbial taxa. By contrast, in ambiguous and wet locations, community assembly more closely followed null patterns. With these results in mind, we next asked what microbial populations in the rhizobiome could contribute to the similarity and stability associated with the dry variety plant hosts.

### Oligotyping analysis revealed selective enrichment of distinct variant compositions within key prokaryotic families in dry rhizobiome communities

To analyze fine-scale ecological patterns in local soil and rhizobiome samples within wet and dry cluster categories, we conducted an unbiased taxonomic oligotype assessment for all ASVs. Consistent with our previous community-level results, our oligotype clustering mirrored ASV clustering, suggesting rare oligotypes had limited influence, while precipitation and ecological pressures drove shared trends between ASVs and their variants across locations (Fig. S4, Table S5).

**Fig 5:**
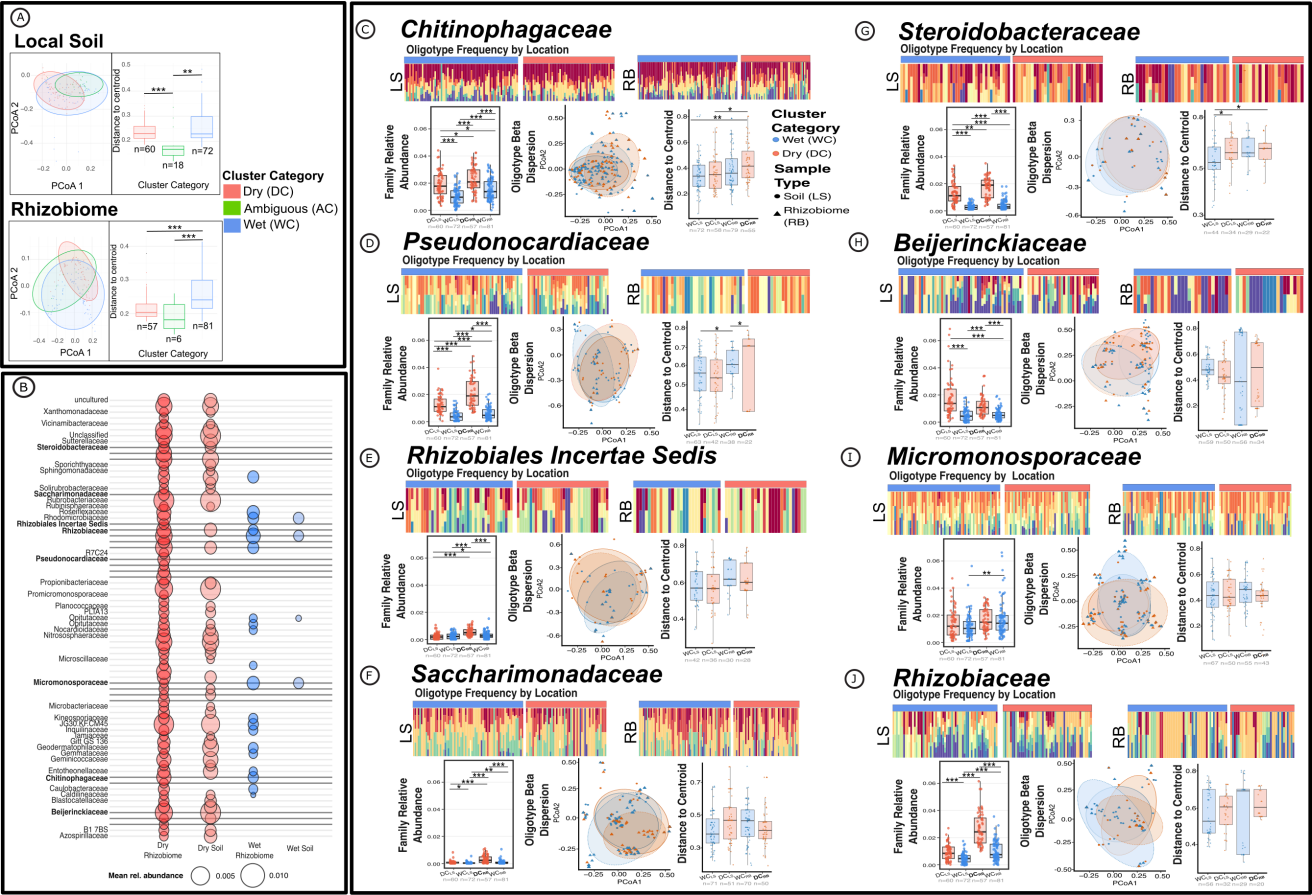
Community variant structure and oligotype composition across cluster category and sample types. (**A**) PCoA plots of beta dispersion based on taxonomically unbiased oligotyping analysis, illustrating community variability within cluster categories for each sample type. Accompanying box plots display the distance of individual samples to their group centroid, describing within-group variability. Statistical significance is denoted as follows: ns = *P* > 0.05, *P < 0.05, **P < 0.01, ***P < 0.001. (**B**) Family indicator taxa analysis showing the mean relative abundance of bacterial families across dry and wet soils and rhizobiomes. (**C–J**) Oligotype-level analyses for representative drought-associated families (*Chitinophagaceae*, *Pseudonocardiaceae*, *Rhizobiales incertae sedis*, *Saccharimonadaceae*, *Steroidobacteraceae*, *Beijerinckiaceae*, *Micromonosporaceae*, and *Rhizobiaceae*). For each family, stacked bar plots show oligotype frequency by location in local soil (LS) and rhizobiome (RB); boxplots depict family-level relative abundance across sample types and cluster categories, where each color corresponds to a distinct oligotype variant; and PCoA ordinations with corresponding beta dispersion quantify variation in oligotype composition among groups. Statistical significance is indicated where applicable as follows: ns = *P* > 0.05, *P < 0.05, **P < 0.01, ***P < 0.001. Details of the oligotypes are in Table S5.

For both sample types, permutation tests for homogeneity of multivariate dispersion revealed significant differences in beta dispersion among our cluster categories (LS and RB: *P* = 0.001; LS: F = 10.9; RB: F = 14.2). However, direct dry-versus-wet comparisons were only significant within the rhizobiome. Local soil communities showed similar distances to centroid between dry and wet groups (0.24 vs 0.26, *P* = 0.13), whereas rhizobiome communities were more dispersed in the wet group than the dry group (0.26 vs 0.21, *P* = 4.25 × 10^−6^) (Fig. 5A). These distinctions likely reflect stronger selection pressures and reduced diversity in drier rhizobiomes. Sharper contrasts between dry soils and rhizobiomes may also arise from host selection (140), microhabitat stabilization (141), and environmental filtering (132).

Given these differences, we focused our oligotyping analysis on enriched ASV families in the dry rhizobiome. Using indicator taxa analysis, we identified families with specific members more abundant in the DC_RB_. We then examined oligotypes within eight of these indicator families with well-documented host-benefiting functions, to test whether drought selected for specific variants in the dry rhizobiome (selected families: *Beijerinckiaceae* [142, 143]*, Chitinophagaceae* [144, 145], *Micromonosporaceae* [146], *Pseudonocardiaceae* [147, 148], *Rhizobiaceae* [149, 150], *Rhizobiales Incertae Sedis* [151, 152], *Saccharimonadaceae* [153], and *Steroidobacteraceae* [154]; *P* < 0.001, log₂ fold change > 1) (Fig. 5B; Table S5). Although exploratory, this analysis was designed to identify candidate lineages for future metagenomic and experimental work.

Taxonomic analysis revealed distinct results across the categorized clusters and sample types among these families (Fig. 5C through J; Table S5). Notably, dry rhizobiomes consistently exhibited clear shifts in within-family oligotype composition. Permutation tests for homogeneity of multivariate dispersion identified significant differences among cluster categories and sample types for *Chitinophagaceae* (*P* = 0.012), *Pseudonocardiaceae* (*P* = 0.020), *Steroidobacteraceae* (*P* = 0.022), *Rhizobiales Incertae Sedis* (*P* = 0.035), and *Saccharimonadaceae* (*P* = 0.047), whereas *Rhizobiaceae* (*P* = 0.297), *Micromonosporaceae* (*P* = 0.348), and *Beijerinckiaceae* (*P* = 0.556) showed no detectable restructuring.

Based on these results, we inferred that some of these families responded to drought through increased abundance, whereas others underwent selective reorganization of variant composition. This pattern is consistent with prior work showing that drought differentially filters rhizosphere taxa by life-history strategy and host association (133, 140, 155). In line with this, families such as *Chitinophagaceae* (156, 157) and *Steroidobacteraceae* (158) include stress-tolerant, resource-specialized lineages likely sensitive to fine-scale environmental and host-mediated selection under water limitation.

Among families with significant differences in dispersion, *Chitinophagaceae* displayed the strongest and most consistent drought-associated separation, indicating pronounced variant turnover specifically within the dry rhizobiome (DC_RB_ vs DC_LS_: 0.415 vs 0.350, p_adj_ = 0.016; DC_RB_ vs WC_LS_: 0.415 vs 0.338, p_adj_ = 0.008). Additionally, *Steroidobacteraceae* showed clear shifts in oligotype composition (DC_RB_ vs WC_LS_: 0.628 vs 0.550, p_adj_ = 0.032; DC_LS_ vs WC_LS_: 0.605 vs 0.550, p_adj_ = 0.042).

We hypothesized that elevated beta dispersion in the DC_RB_ reflected microsite heterogeneity and context-dependent recruitment under extreme drought, where strong filtering constrained the regional species pool; however, local colonization, soil legacy effects, and variable responses generated divergent community configurations. Consistent with our hypothesis, indicator genus-level analysis revealed enriched taxa with known host-benefiting roles within these families in the dry rhizobiome, including *Chitinophaga* (159) and *Niastella* (160) (*Chitinophagaceae*) and *Steroidobacter* (161) (*Steroidobacteraceae*) (*P* < 0.001, log_2_FC > 1). We surmise that these variant shifts likely reflected turnover within these lineages, although our data lacked the resolution for genus-specific oligotype assessment.

Among other families with significant variant beta dispersion differences across categories and sample types, *Pseudonocardiaceae* showed context-dependent oligotype shifts driven by wet samples, with WC_RB_ differing from both DC_LS_ (0.622 vs 0.558, p_adj_ = 0.035) and WC_LS_ (0.622 vs 0.581, p_adj_ = 0.035), while comparisons involving DC_RB_ were not significant (p_adj_ ≥ 0.107). By contrast, *Rhizobiales Incertae Sedis* and *Saccharimonadaceae* showed only weak, non-significant trends after correction, indicating that drought-associated selection on fine-scale diversity was not uniform across enriched families (52, 53, 139).

Notably, several families with the strongest drought-associated increases in relative abundance, particularly Rhizobiaceae, did not exhibit significant oligotype-level restructuring, despite strong enrichment in dry rhizobiomes. We proposed that this decoupling between abundance and oligotype composition indicates that drought responses among enriched families were not uniform, with some taxa responding primarily through expansion of dominant variants, while others exhibited measurable reorganization of within-family variant structure. Our oligotype results suggest that as abiotic stress increased, host influence on rhizobiome assembly also strengthened.

### Conclusion

Precipitation significantly shapes the compositions of microbiome communities (162). However, discrepancies between precipitation and the strength of host interaction can lead to varying impacts on community structure, some of which can be exacerbated under increased abiotic stress (163, 164). Here, we demonstrated that regional precipitation profoundly affected and categorized the microbiomes of *A. gerardii* into two distinct cluster categories (i.e., dry, wet) and one ambiguous cluster category that comprised the intersection between the dry and the wet clusters. Notably, the geographic span of our categorized clusters crossed distinguishable areas of the 100^th^ meridian, known as North America’s “arid-humid divide.” We speculate that the composition and geographic span of our categorized clusters might be linked to the diversity of *A. gerardii* host varieties and legacy effects (9, 12, 165).

Additionally, our findings showed that although pH and precipitation had a more pronounced effect on local soil than on rhizobiome communities, alterations in rhizobiome diversity were more pronounced at precipitation extremes. We surmise that these differences might arise from host variety effects through microhabitat stabilization. By stratifying our samples by type and respective clusters, we further identified large-scale trends in microbial community composition, particularly in the differences between the dry- and wet-categorized clusters. In this study, we observed significant variations in the relative abundances of specific phyla within sample type clusters. This suggests specialized roles of specific phyla responding to precipitation-related stress and underscores potential precipitation-community structure relationships.

**Fig 6:**
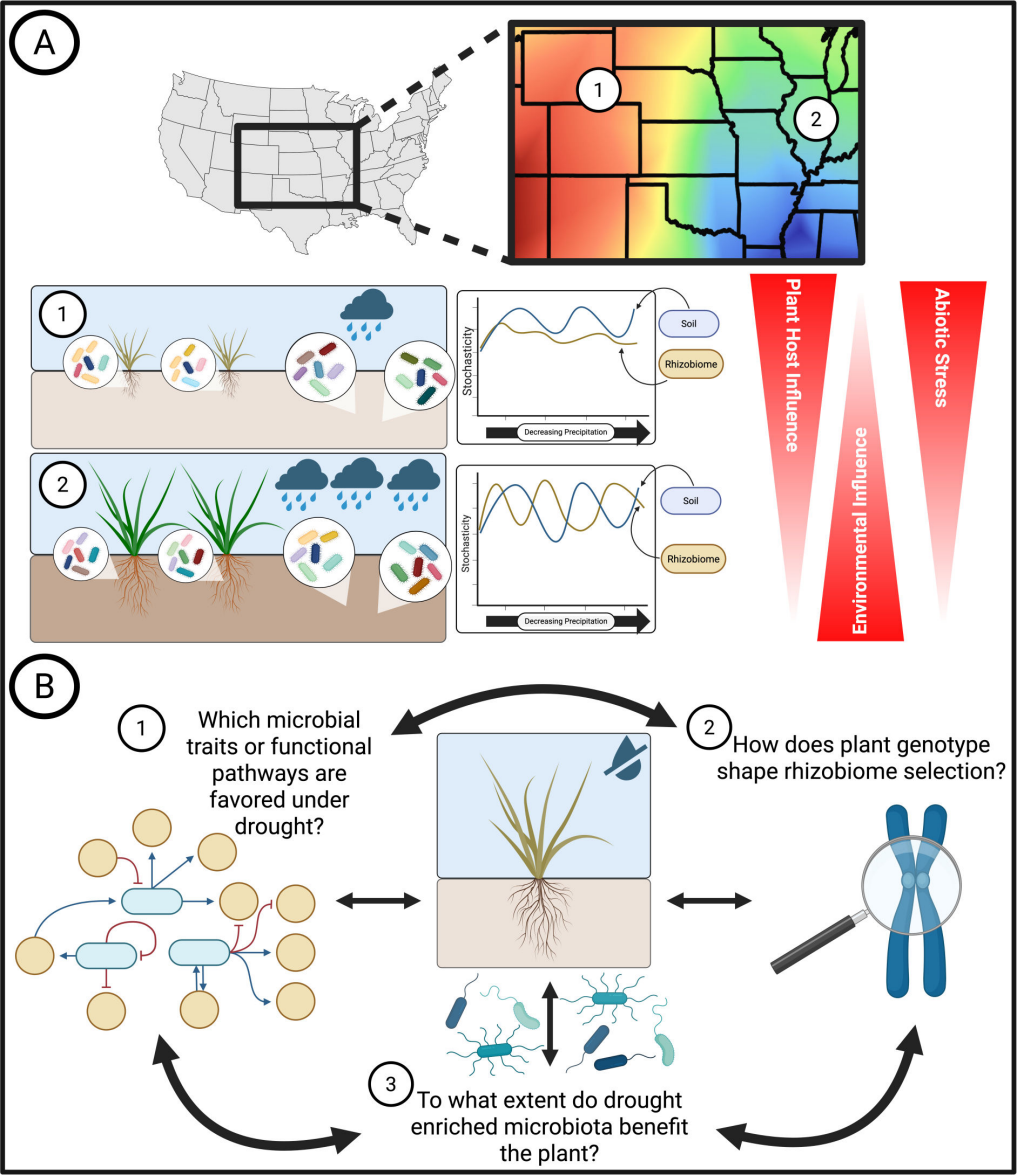
Conceptual model summarizing the influence of precipitation-driven abiotic stress on soil and rhizobiome community assembly. (**A**) The top panel illustrates a subdivision of the U.S. precipitation gradient, highlighting two locations (1 and 2) to describe two scenarios based on our findings. The second panel (location 1) illustrates a high-stress environment with limited precipitation. In this scenario, plant host selection has a strong influence on rhizobiome community assembly, leading to divergence from the surrounding soil community and the enrichment of specialist taxa. This shift reflects a reduction in environmental influence and an increase in host-mediated filtering under abiotic stress. Here, the stochasticity of the rhizobiome becomes more uniform, with more similar plant-associated rhizobiome compositions, as site aridity increases, while the local soil remains highly variable. In contrast, the bottom panel (location 2) depicts a low-stress environment characterized by high precipitation. Under these conditions, environmental influences dominate, while the impact of plant hosts is minimal. Consequently, local soil and rhizobiome communities exhibit high variability. Generalist taxa dominate both communities. Here, the stochasticity of the local soil and rhizobiome remains high, showing a highly variable plant-associated rhizobiome, and precipitation has a minimal effect. (**B**) Building on these results, the figure outlines three questions guiding future work: (1) which microbial traits or pathways are favored by host filtering under drought; (2) how plant genotype and local soil conditions shape host-driven rhizobiome selection during water limitation; and (3) whether drought-enriched microbes enhance plant fitness or stress tolerance.

Furthermore, we showed notable differences in rhizobiome and local soil microbial diversity with reduced precipitation, revealing possible links between abiotic stress and inferred microbial community assembly strategies (stochastic vs deterministic). We further demonstrated that regional precipitation was a critical factor in rhizobiome assembly, with the rhizosphere communities in the aridest locations exhibiting more predicted forms of randomness than the local soil, implying host-associated biotic filtering under extreme abiotic stress (Fig. 6A). Our findings highlight the shift toward reduced stochasticity in the microbial communities under intensified abiotic stress, prompting several key questions that will guide future directions of this work: (i) which microbial traits or functional pathways are selectively favored by host-mediated filtering under drought conditions; (ii) to what extent plant genotype and local soil context modulate host-driven selection of the rhizobiome under water limitation; and (iii) whether drought-enriched microbial lineages confer measurable fitness or stress-tolerance benefits to the plant host (Fig. 6B). This study underscores the pivotal role of regional precipitation in shaping plant-associated rhizobiomes and suggests that plant hosts might rely heavily on biotic filtering mechanisms to mitigate extreme environmental challenges, providing a framework for future work aimed at resolving the specific microbial traits, interactions, and functional strategies that underlie drought resilience in tallgrass prairies.

## Data Availability

All raw sequence data for this study are publicly available in the NCBI SRA under BioProject accession number PRJNA1249957. All bioinformatic and R codes are deposited in GitHub (https://github.com/SonnyTMLee/Plant-host-identity-drives-rhizobiome-assembly-under-increasing-abiotic-stress.git).

## SUPPLEMENTAL MATERIAL

Figure S1 spectrum.02832-25-s0001.pdf Microbial community composition across climate and soil property gradients.

Figure S2 spectrum.02832-25-s0002.pdf Linear regression analysis of phylum-level microbial abundance across categories and sample types with increasing precipitation depth.

Figure S3 spectrum.02832-25-s0003.pdf Linear regression analysis of phylum-level microbial abundance across categories and sample types with increasing precipitation depth.

Figure S4 spectrum.02832-25-s0004.pdf Taxonomically unbiased oligotype clustering.

Supplemental legends spectrum.02832-25-s0005.docx Descriptive legends for all supplemental material.

Table S1 spectrum.02832-25-s0006.xlsx Site locations with precipitation metrics.

Table S2 spectrum.02832-25-s0007.xlsx QIIME local soil and rhizobiome sequences before and after quality trimming; ASV assignments for the local soil and rhizobiome samples.

Table S3 spectrum.02832-25-s0008.xlsx Soil chemistry, soil properties, and alpha diversity statistical analyses, alpha diversity for the local soil and rhizobiome samples, and precipitation and pH gradient t-test results.

Table S4 spectrum.02832-25-s0009.xlsx ASV distribution by sample type and cluster category.

Table S5 spectrum.02832-25-s0010.xlsx Beta dispersion distance, RCBray, and beta-NTI metric for local soil and rhizobiome samples within a location; indicator taxa; and unbiased and biased taxonomic oligotyping results.

ASM does not own the copyrights to Supplemental Material that may be linked to, or accessed through, an article. The authors have granted ASM a non-exclusive, world-wide license to publish the Supplemental Material files. Please contact the corresponding author directly for reuse.
