# Supplementary material for: Plant influence shapes Andropogon gerardii rhizobiome assembly strategies in response to decreasing precipitation
Source: Microbiol Spectr. 2026 Jul 15;14(8):e02832-25. doi: 10.1128/spectrum.02832-25 (PMC13435935; doi:10.1128/spectrum.02832-25)
Supplement: Supplementary file 1 [file spectrum.02832-25-s0001.pdf]

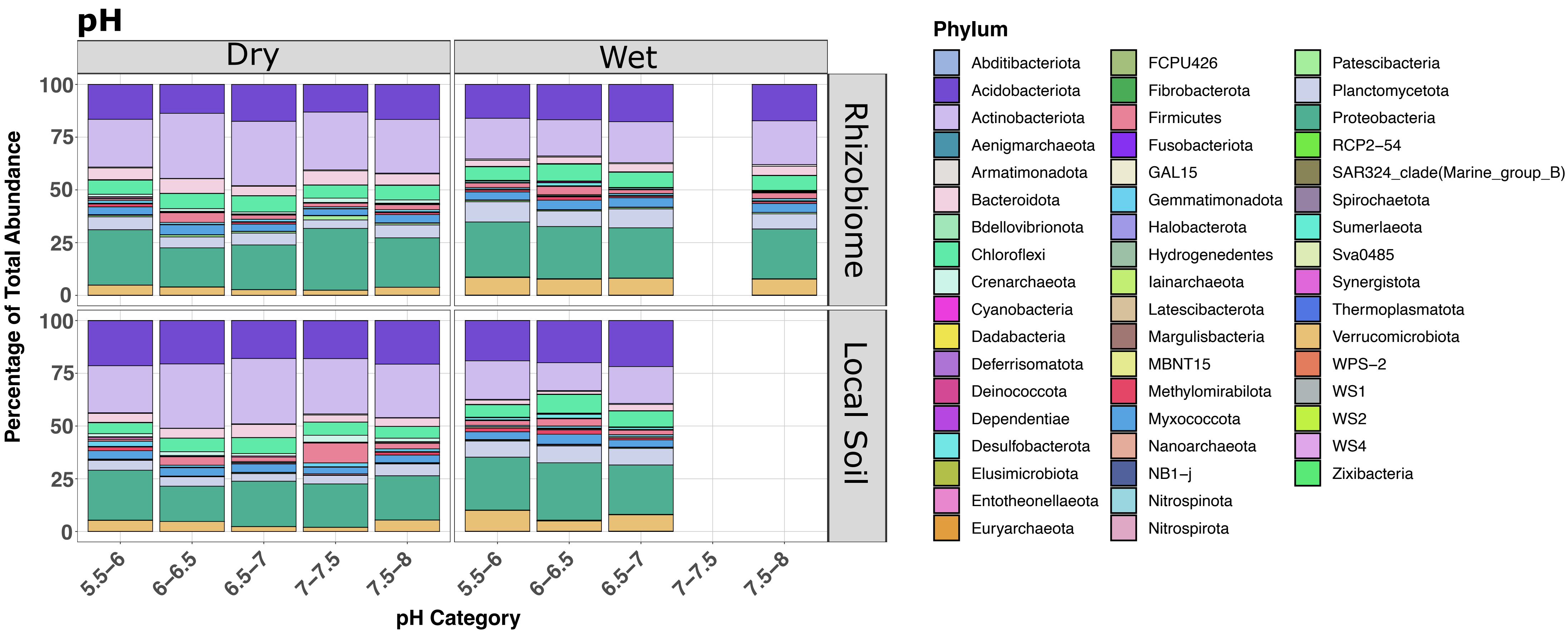

pH
Dry
Wet
Rhizobiome
Local Soil
Percentage of Total Abundance
0
25
50
75
100
5.5–6
6–6.5
6.5–7
7–7.5
7.5–8
pH Category
Phylum
Abditibacteriota
Acidobacteriota
Actinobacteriota
Aenigmarchaeota
Armatimonadota
Bacteroidota
Bdellovibrionota
Chloroflexi
Crenarchaeota
Cyanobacteria
Dadabacteria
Deferrisomatota
Deinococcota
Dependentiae
Desulfobacterota
Elusimicrobiota
Entotheonellaeota
Euryarchaeota
FCPU426
Fibrobacterota
Firmicutes
Fusobacteriota
GAL15
Gemmatimonadota
Halobacterota
Hydrogenedentes
Iainarchaeota
Latescibacterota
Margulisbacteria
MBNT15
Methylomirabilota
Myxococcota
Nanoarchaeota
NB1−j
Nitrospinota
Nitrospirota
Patescibacteria
Planctomycetota
Proteobacteria
RCP2−54
SAR324_clade(Marine_group_B)
Spirochaetota
Sumerlaeota
Sva0485
Synergistota
Thermoplasmatota
Verrucomicrobiota
WPS−2
WS1
WS2
WS4
Zixibacteria

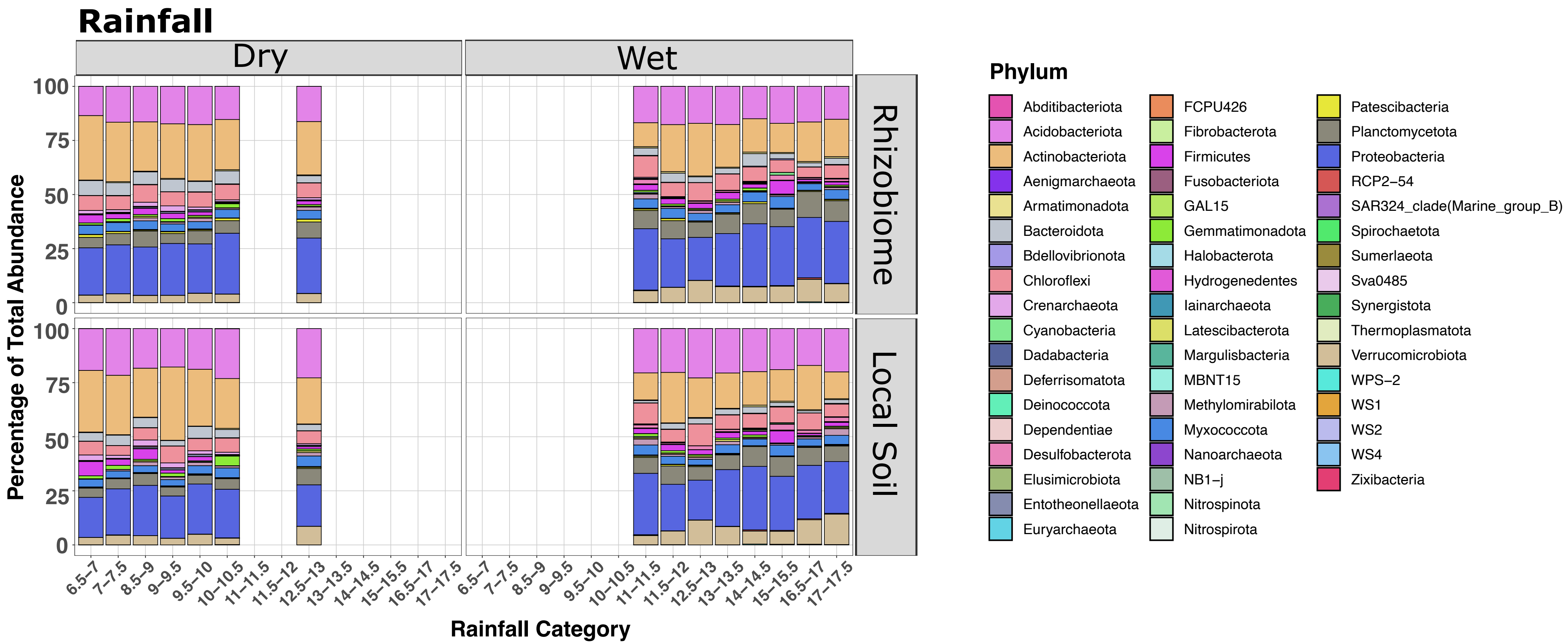

Rainfall
Dry
Wet
Rhizobiome
Local Soil
Percentage of Total Abundance
0
25
50
75
100
6.5–7
7–7.5
8.5–9
9–9.5
9.5–10
10–10.5
11–11.5
11.5–12
12.5–13
13–13.5
14–14.5
15–15.5
16.5–17
17–17.5
Rainfall Category
Phylum
Abditibacteriota
Acidobacteriota
Actinobacteriota
Aenigmarchaeota
Armatimonadota
Bacteroidota
Bdellovibrionota
Chloroflexi
Crenarchaeota
Cyanobacteria
Dadabacteria
Deferrisomatota
Deinococcota
Dependentiae
Desulfobacterota
Elusimicrobiota
Entotheonellaeota
Euryarchaeota
FCPU426
Fibrobacterota
Firmicutes
Fusobacteriota
GAL15
Gemmatimonadota
Halobacterota
Hydrogenedentes
Iainarchaeota
Latescibacterota
Margulisbacteria
MBNT15
Methylomirabilota
Myxococcota
Nanoarchaeota
NB1−j
Nitrospinota
Nitrospirota
Patescibacteria
Planctomycetota
Proteobacteria
RCP2−54
SAR324_clade(Marine_group_B)
Spirochaetota
Sumerlaeota
Sva0485
Synergistota
Thermoplasmatota
Verrucomicrobiota
WPS−2
WS1
WS2
WS4
Zixibacteria
