# Supplementary material for: Plant influence shapes Andropogon gerardii rhizobiome assembly strategies in response to decreasing precipitation
Source: Microbiol Spectr. 2026 Jul 15;14(8):e02832-25. doi: 10.1128/spectrum.02832-25 (PMC13435935; doi:10.1128/spectrum.02832-25)
Supplement: Supplementary file 2 [file spectrum.02832-25-s0002.pdf]

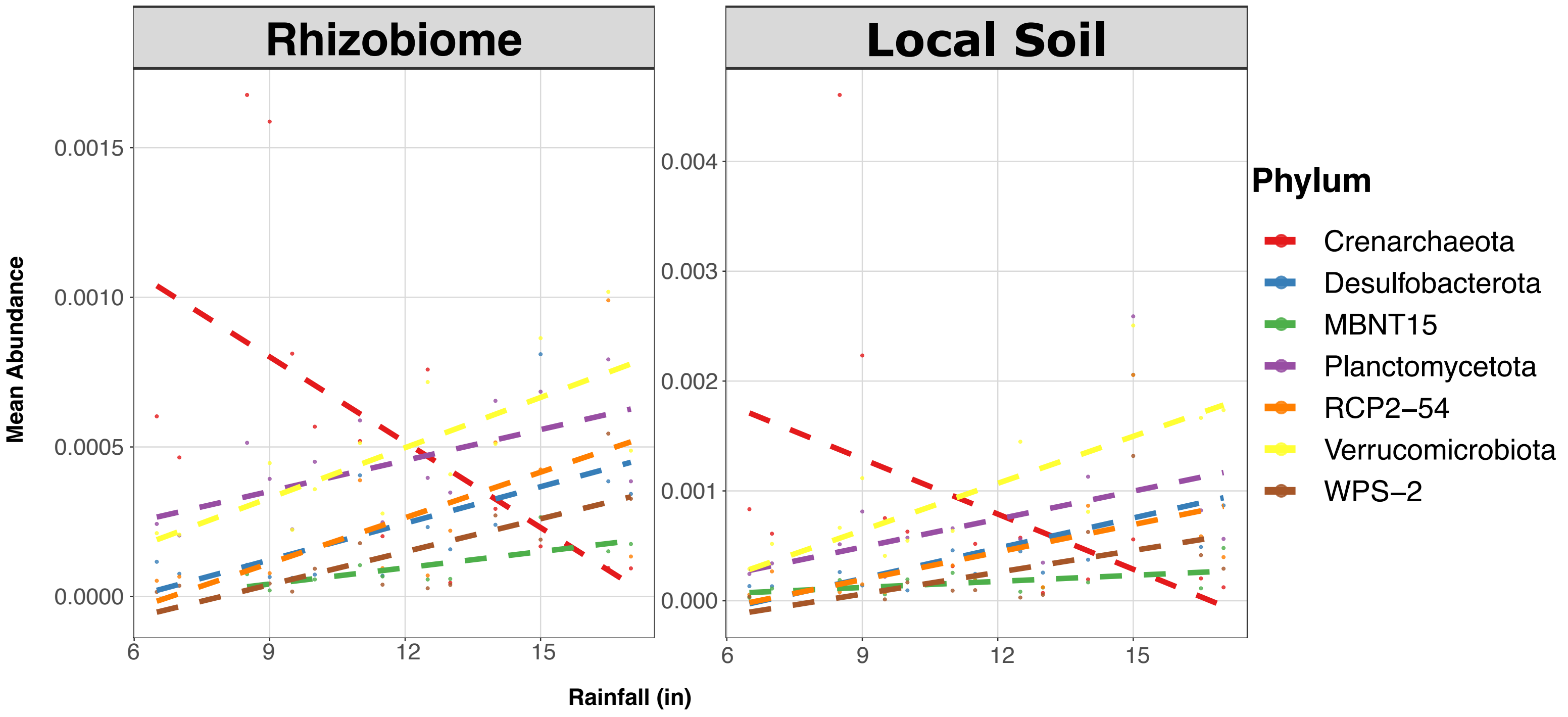
Rhizobiome
Local Soil
Mean Abundance
0.0015
0.0010
0.0005
0.0000
0.004
0.003
0.002
0.001
0.000
6
9
12
15
Rainfall (in)
Phylum
Crenarchaeota
Desulfobacterota
MBNT15
Planctomycetota
RCP2−54
Verrucomicrobiota
WPS−2
