# Supplementary material for: Plant influence shapes Andropogon gerardii rhizobiome assembly strategies in response to decreasing precipitation
Source: Microbiol Spectr. 2026 Jul 15;14(8):e02832-25. doi: 10.1128/spectrum.02832-25 (PMC13435935; doi:10.1128/spectrum.02832-25)
Supplement: Supplementary file 3 [file spectrum.02832-25-s0003.pdf]

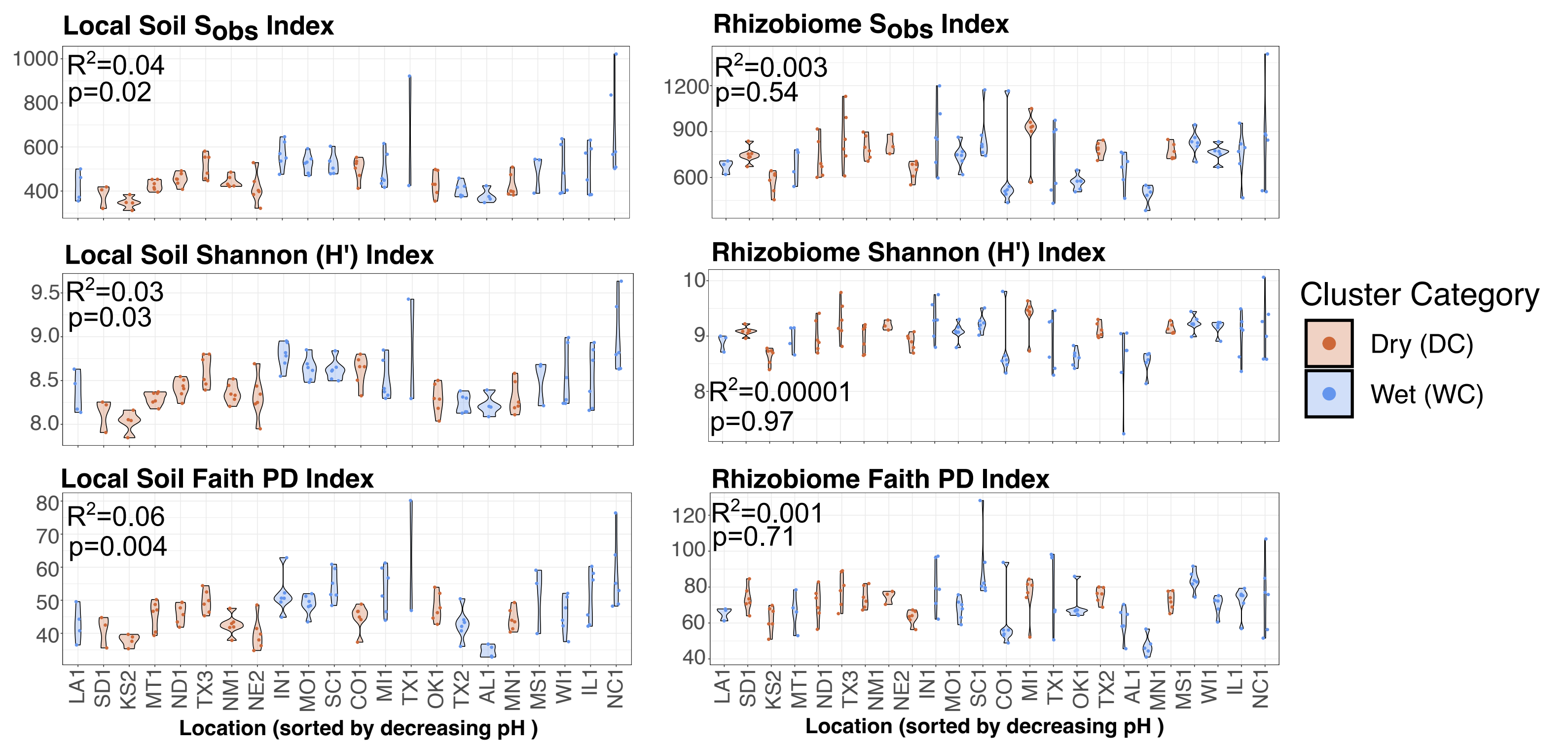

Local Soil Sobs Index
R2=0.04
p=0.02
Rhizobiome Sobs Index
R2=0.003
p=0.54
Local Soil Shannon (H') Index
R2=0.03
p=0.03
Rhizobiome Shannon (H') Index
R2=0.00001
p=0.97
Local Soil Faith PD Index
R2=0.06
p=0.004
Rhizobiome Faith PD Index
R2=0.001
p=0.71
Cluster Category
Dry (DC)
Wet (WC)
LA1 SD1 KS2 MT1 ND1 TX3 NM1 NE2 IN1 MO1 SC1 CO1 MI1 TX1 OK1 TX2 AL1 MN1 MS1 WI1 IL1 NC1
Location (sorted by decreasing pH)
