# Supplementary material for: Plant influence shapes Andropogon gerardii rhizobiome assembly strategies in response to decreasing precipitation
Source: Microbiol Spectr. 2026 Jul 15;14(8):e02832-25. doi: 10.1128/spectrum.02832-25 (PMC13435935; doi:10.1128/spectrum.02832-25)
Supplement: Supplementary file 4 [file spectrum.02832-25-s0004.pdf]

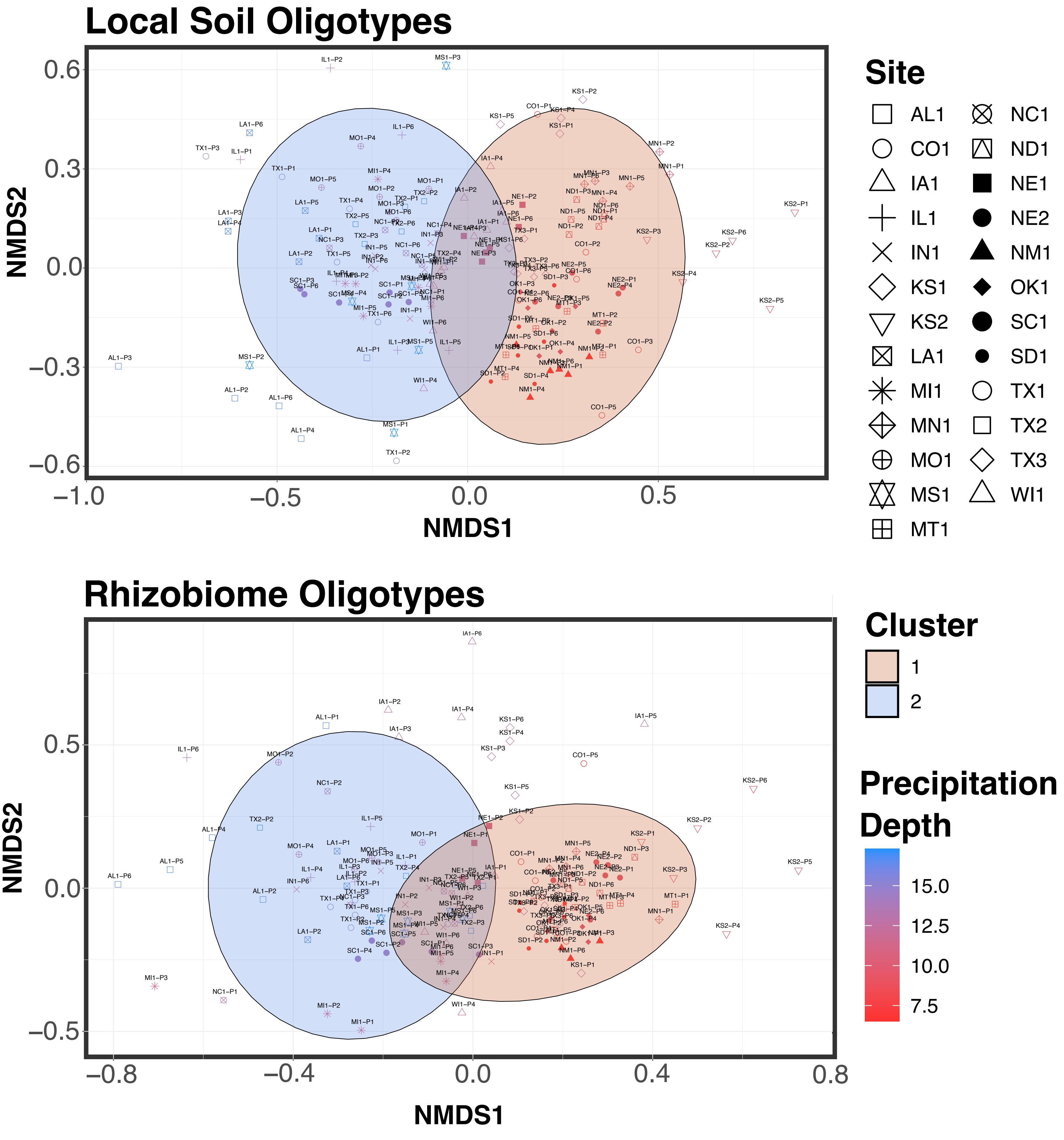
Local Soil Oligotypes
NMDS2
0.6
0.3
0.0
−0.3
−0.6
−1.0
−0.5
0.0
0.5
NMDS1
Site
AL1
CO1
IA1
IL1
IN1
KS1
KS2
LA1
MI1
MN1
MO1
MS1
MT1
NC1
ND1
NE1
NE2
NM1
OK1
SC1
SD1
TX1
TX2
TX3
WI1
Rhizobiome Oligotypes
NMDS2
0.5
0.0
−0.5
−0.8
−0.4
0.0
0.4
0.8
NMDS1
Cluster
1
2
Precipitation Depth
15.0
12.5
10.0
7.5
