# Supplementary material for: Plant influence shapes Andropogon gerardii rhizobiome assembly strategies in response to decreasing precipitation
Source: Microbiol Spectr. 2026 Jul 15;14(8):e02832-25. doi: 10.1128/spectrum.02832-25 (PMC13435935; doi:10.1128/spectrum.02832-25)
Supplement: Supplementary file 5 [file spectrum.02832-25-s0005.docx]

**Supplementary Figures**

**Supplementary** **Figure S1: *Microbial community composition across climate and soil property gradients***

Stacked bar plots showing the relative abundance of ASVs classified at the phylum level, stratified by cluster category, environmental pH categories (top), and precipitation (bottom) gradients. This visualization highlights compositional shifts in microbial communities along gradients of climate and soil properties.

**Supplementary** **Figure S2: *Linear regression analysis of phylum-level microbial abundance across categories and sample types with increasing precipitation depth***

Linear models assessed phyla whose relative abundances significantly correlated with precipitation across different categories and sample types (local soil and rhizobiome). Only phyla exhibiting significant relationships with precipitation (p < 0.05) are shown, highlighting taxa potentially responsive to moisture variability along the precipitation gradient.

**Supplementary** **Figure S3: *pH linear regression analysis on alpha diversity***

Linear regression analysis was conducted to evaluate the relationship between pH and alpha diversity metrics, including S_obs_, H', and Faith PD, in local soil (left panels) and rhizobiome (right panels) communities. Samples were arranged along a gradient of decreasing pH and visualized using violin plots. The plot color denotes the location's cluster category (dry and wet). Displayed in each of the plots are the p and R^2^ values from the regression analysis.

**Supplementary** **Figure S4: *Taxonomically unbiased oligotype clustering***

NMDS taxonomically unbiased oligotype clustering of local soil (top) and rhizobiome (bottom) variants within each sample with k-means clustering and optimized silhouette scores showing distinct groupings with 95% confidence ellipses. Each point represents a sample, colored according to NOAA's 60-day maximum precipitation data at each site.

**Supplementary Tables**

**Supplementary Table S1:** Site Locations with Precipitation Metrics

**Supplementary Table S2:** QIIME Local Soil and Rhizobiome Sequences Before and After Quality Trimming; ASV Assignments for the Local Soil and Rhizobiome Samples

**Supplementary Table S3:** Soil Chemistry; Soil Properties and Alpha Diversity Statistical Analyses; Alpha Diversity for the Local Soil and Rhizobiome Samples; Precipitation and pH Gradient t-Test Results

**Supplementary Table S4:** ASV Distribution by Sample Type and Cluster Category

**Supplementary Table S5:** Beta Dispersion Distance, RC_Bray,_ and βNTI Metric for Local Soil and Rhizobiome Samples within a Location; Indicator Taxa; Unbiased and Biased Taxonomic Oligotyping Results
